# Ultrathin Covalent Organic Framework Membranes with Tailorable Porous Channels for High-Permeance Helium Separation

**DOI:** 10.1021/jacs.6c04426

**Published:** 2026-06-04

**Authors:** Wei Xie, Fengxiang Zhao, Tengyang Zhu, Chao Sun, Jichao Zhang, Renhao Dong

**Affiliations:** † Key Laboratory of Colloid and Interface Chemistry, School of Chemistry and Chemical Engineering, 12589 Shandong University, Jinan 250100, China; ‡ Department of Chemistry, 25809 The University of Hong Kong, Hong Kong 999077, China; § Materials Innovation Institute for Life Sciences and Energy (MILES), HKU-SIRI, Shenzhen 518048, China; ∥ Shanghai Synchrotron Radiation Facility, Shanghai Advanced Research Institute, 202002 Chinese Academy of Sciences, Shanghai 201204, China

## Abstract

Covalent organic frameworks (COFs) are a unique class of porous crystalline materials featuring precisely ordered structures and well-tunable porous channels as well as abundant active functional groups, which enable them as promising membranes for high-performance gas separation. However, construction of thin COF membranes to simultaneously realize high-permeability and high-selectivity He separation has remained a challenge. Here, we report the synthesis of ultrathin (<5 nm thick) porphyrinyl two-dimensional polyimine (Por-2DPI) COF membranes, which comprise single-crystalline domains and highly ordered, vertically aligned one-dimensional (1D) porous channels. We further develop an inner-pore coordination (IPC) strategy to graft bulky phenyl (Ph) and naphthyl (Np) groups on the inner walls of the porous channels. This strategy effectively narrows the intrinsic pore size from ∼2.0 to ∼1.4 nm, which leads to the efficient tailoring of the transmembrane transport toward the high-permeance He separation with high selectivity. Notably, the resultant Por-2DPI-Np COF membrane simultaneously delivers a record-high He permeance of 4323 GPU and a high selectivity of 77.4 (He/CH_4_), superior to the state-of-the-art reported membranes. The contrast experiments supported by theoretical modeling reveal that the exceptional performance arises from the synergistic effect, where the ultrathin feature and 1D pore arrays ensure high permeance. Meanwhile, the grafted branches impede the diffusion of CH_4_ more significantly than that of He, resulting in an enhanced molecular sieving effect for high selectivity. Our work provides a general strategy to overcome the permeability–selectivity trade-off and highlights the unprecedented potential of engineered COF membranes for highly efficient helium recovery.

## Graphical abstract

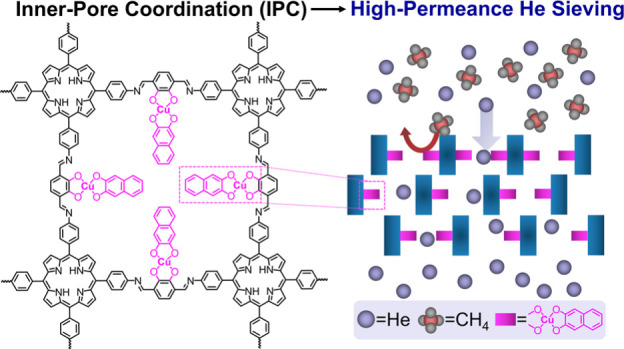

## Introduction

Helium (He) is a high-value inert gas widely used in fields such as electronics, fiber optics, aerospace, and healthcare.^1^ Due to the rising global demand, the efficient separation of He from natural gas streams—especially from methane (CH_4_) and nitrogen (N_2_)—has become increasingly important.^2^ Membrane-based separation stands out as an ideal technology due to its inherent advantages of high energy efficiency, operational simplicity, and scalability.^3–5^ Even though significant research efforts have been dedicated to developing advanced membrane materials, evolving from traditional polymers to microporous materials,^6^ these reported membranes still face many limits. Crystalline porous materials such as zeolites and metal–organic frameworks (MOFs) are highly tunable, making them well-suited for gas separations with high selectivity. Nevertheless, both zeolite and MOF-based membranes continue to face intrinsic limitations from grain boundary defects, and their large-scale production remains nontrivial.^7,8^ Linear polymer membranes, while more processable, usually suffer from a trade-off between permeability and selectivity,^9,10^ making it difficult to simultaneously achieve the high throughput and precise molecular sieving required for economical He extraction.^11,12^ Therefore, there is an urgent need to develop efficient strategies for tailoring the porous channels of membranes that enable high-permeance and highly selective He separation.

Two-dimensional covalent organic frameworks (2D COFs)^13–15^ are a class of layer-stacked porous crystalline materials, featuring high thermochemical stability, ordered porous channels, structural designability and high specific surface area, etc., which have been considered to be promising gas separation materials.^16–18^ While COF-based membranes have achieved remarkable success in the selective separation of different gas molecules (e.g., CO_2_ separation and H_2_ purification),^19–21^ their exploration in high-precision He separation remains extremely scarce. The intrinsic pore apertures of most 2D COFs (usually >0.6 nm) are substantially larger than the kinetic diameters of key gas molecules involved in He purification (He: ∼ 0.26 nm; CH_4_: ∼ 0.38 nm; N_2_: ∼ 0.36 nm). This mismatch leads to limited size-sieving selectivity, as gases primarily separate via Knudsen diffusion rather than molecular sieving.^22^ Consequently, precise pore engineering is imperative to shrink these channels to a size relevant for He sieving.^23^

The reported strategies, such as staggered interlayer stacking or fillers incorporation, attempt to solve this by aggressively reducing the effective pore size.^19–21,23^ Yet, executing such drastic spatial compression inevitably triggers a punishing permeability–selectivity trade-off. Given the scarcity of COF-based He separation, the severity of this structural dilemma is best illustrated by the broader landscape of state-of-the-art He separation membranes, including metal–organic frameworks (MOFs) and advanced polymers.^6,24,25^ Across these systems, achieving enhanced He/CH_4_ selectivity (typically reaching 20–50) inevitably involves a severe trade-off, leading to a significant increase in diffusion resistance and a consequent reduction in gas permeance (frequently falling to a few hundred GPU or even lower).^1,4,6^ This trade-off is particularly acute for He separation, where the target He molecule is the smallest, demanding extreme precision in pore narrowing without sacrificing the ultrathin, fast-transport pathways.^26^ Therefore, simultaneously achieving precise pore constriction and maintaining the high-flux structural features (i.e., an ultrathin morphology, face-on orientation, and continuous 1D channels) remains a significant hurdle, hindering the development and leading to the scarcity of high-performance COF-based membranes for He/CH_4_ and He/N_2_ separation.

Here, we address the above challenge by developing an efficient inner-pore coordination (IPC) approach for tailoring the porous channels of COF membranes, leading to the exceptionally high-performance He selective separation. We synthesized an ultrathin (<5 nm) porphyrin-based 2D polyimine (Por-2DPI) COF membrane on the water surface,^27–29^ which features single-crystalline domains with a face-on orientation and vertically aligned one-dimensional (1D) channels, providing extremely high He permeance (39262 GPU). Subsequently, we developed an IPC strategy to precisely graft bulky aryl groups (including phenyl (Ph) and naphthyl (Np) groups) within the 1D porous channels, thus generating Por-2DPI-Ph and Por-2DPI-Np COF membranes. This in situ modification effectively tailors the pore size (from 2.2 to 1.7 and 1.3 nm, respectively) and chemical environment without compromising the structural integrity. Notably, the resultant assembled Por-2DPI-Np membrane delivered outstanding He permeance reaching 4323 GPU, coupled with high He/CH_4_ and He/N_2_ selectivities of 77.4 and 52.3, respectively. These values surpass those of the state-of-the-art membranes and are 2 orders of magnitude higher in permeance than commercial linear polymeric membranes. The exceptional performance is attributed to a synergistic mechanism. The ultrathin nature and face-on orientation of the continuous channels ensure a fundamentally high He permeance. Concurrently, the narrowed pore sizes and coordinated molecular barriers induce strong steric hindrance. This heavily suppresses the diffusion of larger gas molecules (both CH_4_ and N_2_, which possess similarly large kinetic diameters), while allowing the small He molecules to pass unhindered, thereby generating a profound differential diffusion inhibition. Our work provides a versatile pore-engineering strategy applicable to 2D framework membrane materials, opening new pathways for designing high-performance He separation membranes.

## Results and Discussion

### Synthesis and Characterization of 2DPI COF Membranes

The Por-2DPI membranes were synthesized on the water surface assisted by a surfactant monolayer (Figures 1a,b and S1).^28,30^ In a typical synthetic procedure, a sodium (9Z)-octadec-9-en-1-yl sulfate (sodium oleyl sulfate, SOS) monolayer was prepared on the water surface, and then an aqueous solution of TAPP (4,4’,4’’,4’’’-(21H,23H-porphyrin-5,10,15,20-tetryl)­tetraaniline) protonated with TfOH was added into the subphase. After the coassembly of SOS and TAPP on the water surface, the aqueous solution of 2,3-DHTA (2,3-dihydroxybenzene-1,4-dicarbaldehyde) containing adjacent hydroxyl groups was injected into the water phase to trigger the condensation polymerization underneath the surfactant monolayer. A highly crystalline Por-2DPI membrane was obtained after 5 days of reaction at 50 °C, and then horizontally transferred onto different substrates (such as Si/SiO_2_, copper foil, and PAN) for further characterization and separation tests (the detailed transfer procedure is provided in the Supporting Information). Digital photographs confirm that the membrane remains macroscopically intact both at the air–water interface (6 cm diameter) and following transfer onto centimeter-scale substrates (Figure S2). Meanwhile, optical microscopy further verifies its continuous and defect-free nature at the microscopic scale (Figure 1c). The atomic force microscopy (AFM) measurements indicated the thickness of the as-prepared Por-2DPI membrane as low as 4 nm (Figure 1d), which could also be tuned up to ∼ 200 nm via varying the monomer concentration from 0.08 μmol to 1.50 μmol (the detailed morphological and roughness evolution associated with the island growth and coalescence mechanism is provided in Figure S3).

**Figure 1:**
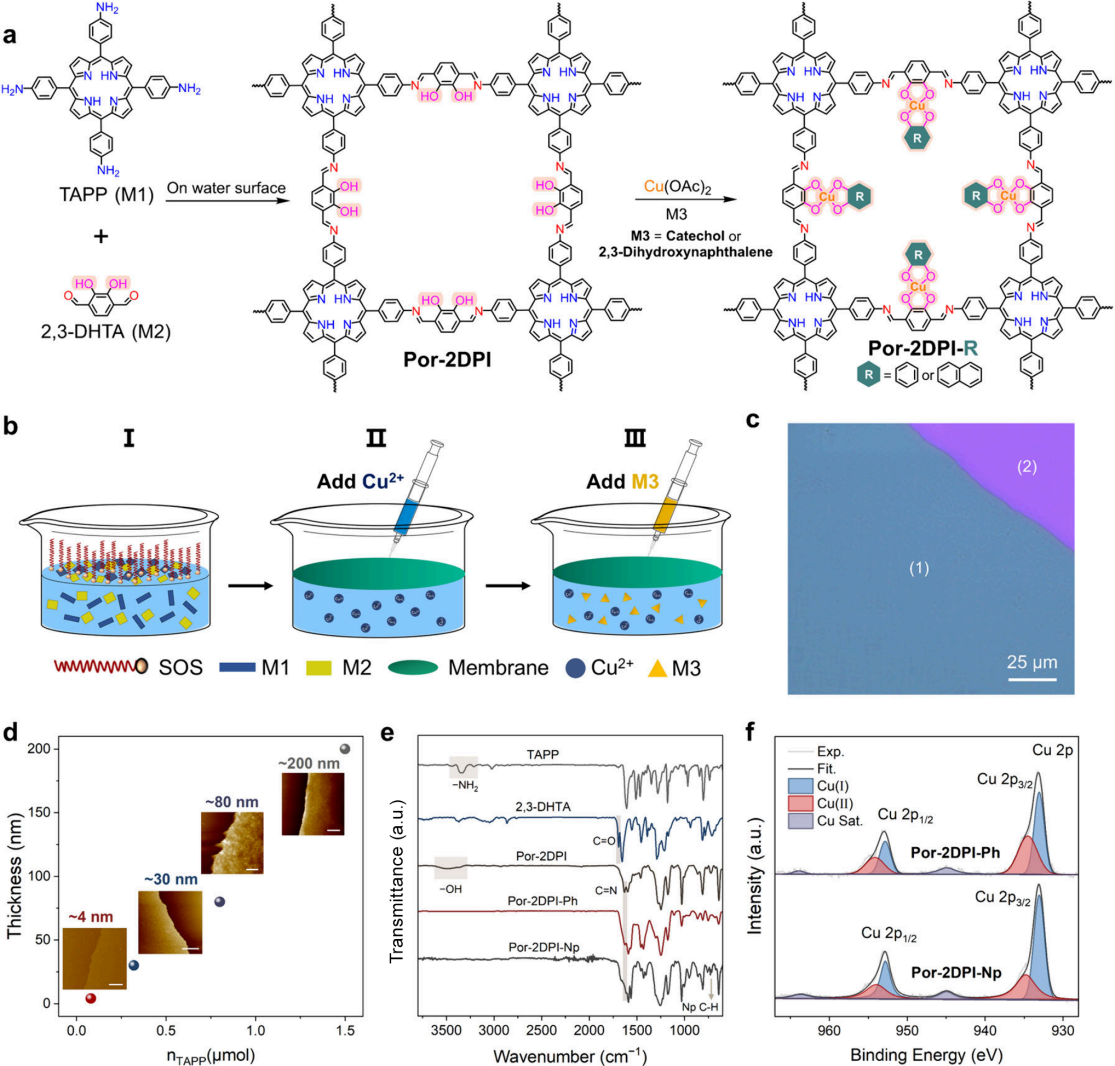
(a and b) Schematic illustration of the chemical structures and the on-water synthesis procedure of the targeted Por-2DPI and Por-2DPI-R (R = Ph or Np) COF membranes, respectively. (c) Optical microscopy image of Por-2DPI membrane, where (1) and (2) represent membrane and SiO_2_ substrate, respectively. (d) AFM images and relevant thickness changes of Por-2DPI membranes with varying the concentration of TAPP: 4 nm (0.08 μmol), 30 nm (0.32 μmol), 80 nm (0.80 μmol), 200 nm (1.50 μmol). Scale bars: 2 μm. (e) FTIR-ATR spectra of precursor monomers and COF membranes. (f) High-resolution XPS spectra of Cu 2p in Por-2DPI-Ph and Por-2DPI-Np.

Following the formation of Por-2DPI COF membranes, the acidic subphase was removed and replaced by neutral water (Figure 1b). Then, the Cu­(OAc)_2_ aqueous solution (5 mg mL^–1^, 1 mL) and the NaOAc aqueous solution (2 mg mL^–1^, 1 mL) were injected into the aqueous phase, and the aqueous solution of aryl monomers such as catechol and 2,3-dihydroxynaphthalene (2 mg mL^–1^, 5 mL) was also injected. Finally, the Por-2DPI-R (R = Ph or Np) COF membranes were achieved after 24 h at room temperature via the IPC strategy. The detailed optimization of these coordination conditions including the reaction time and temperature is provided in the Supporting Information (Figure S4).

The Raman spectra (Figure S5) show that the characteristic −NH_2_ stretching peak of TAPP at 1269 cm^–1^ disappears in the resultant Por-2DPI and Por-2DPI-R membranes, while the appearance of a new C=N peak (1665 cm^–1^) indicates the conversion of amine monomers into imine polymers. We further employed ex-situ FT-IR to probe the structural evolution (Figure 1e). The characteristic C=O stretching of 2,3-DHTA at 1694 cm^–1^ disappears in Por-2DPI and Por-2DPI-R membranes, and a new peak C=N (1630 cm^–1^) appears, which proves the conversion of aldehyde monomers to imine polymers. Additionally, the drastic attenuation of the hydroxyl stretches around 3450 cm^–1^ in Por-2DPI-Ph and Por-2DPI-Np provided strong evidence for the highly efficient coordination with copper ions. This was further corroborated by the emergence of a characteristic out-of-plane C–H bending peak at ∼ 720 cm^–1^ in the Por-2DPI-Np spectrum, firmly verifying the successful anchoring of the naphthyl groups.

The successful implementation of the IPC strategy and the chemical state of copper in the modified Por-2DPI-R membranes were confirmed by X-ray photoelectron spectroscopy (XPS). As shown in Figure S6, the survey spectra clearly indicate the absence of Cu signals in the pristine Por-2DPI, whereas distinct Cu 2p peaks emerge in both Por-2DPI-Ph and Por-2DPI-Np, providing direct evidence for the introduction of copper species through the IPC process. High-resolution XPS analysis of the Cu 2p region (Figure 1f) displays the characteristic spin–orbit doublet (Cu 2p_3/2_ and Cu 2p_1/2_ at ∼ 933.0 and ∼ 952.9 eV), verifying the successful coordination of copper ions within the framework. To quantitatively evaluate the efficiency of the IPC modification, the coordination degree was estimated by comparing the experimental atomic ratio of Cu to N (Cu/N)_*exp*_ obtained from XPS with the theoretical ratio (Cu/N)_*theo*_ derived from the ideal molecular structure (where all pore sites are occupied). The calculated coordination ratios reach approximately 87% for Por-2DPI-Ph and 92% for Por-2DPI-Np. These high values demonstrate not only the successful grafting of Cu^2+^-aryl complexes within the 1D porous channels but also the remarkable efficiency of our IPC strategy in achieving precise pore functionalization.

### Crystalline Structures of 2DPI COF Membranes

Grazing-incidence wide-angle X-ray scattering (GIWAXS) was employed to elucidate the molecular packing and orientation of the transferred membranes. As depicted in Figure 2a, the pristine Por-2DPI exhibits sharp in-plane *Q*_*xy*_ Bragg peaks at 0.25, 0.50, 0.75, and 1.00 Å^–1^, indexing to the (100), (200), (300), and (400) reflections, respectively. This profile translates to a square lattice with *a* = *b* = 25.3 Å (Figures 2c and S7a,b). Along the out-of-plane direction, a broad halo at *Q*_*z*_ = 1.60 Å^–1^ is assigned to the (001) reflection, indicating an average interplanar *d*-spacing of ∼ 3.9 Å driven by the noncovalent layer stacking. These scattering signatures confirm a strict face-on orientation of the Por-2DPI channels relative to the substrate. Following the IPC modification, the Por-2DPI-Np membrane retains the foundational square lattice but exhibits specific scattering intensity modulations (Figure 2b,c). Notably, a distinct new peak emerges at *Q*_*xy*_ = 0.36 Å^–1^, corresponding to the (110) plane. The appearance of this reflection indicates a shift in the structure factor, suggesting an ordered spatial arrangement of the grafted naphthyl groups within the pores. Concurrently, the primary (100) reflection and higher-order reflections along the *h*00 axis (e.g., (400) and (500)) suffer a severe drop in intensity. This selective attenuation is a classic hallmark of the pore-filling effect rather than a loss of crystallinity: the introduction of dense aromatic rings and copper centers into the channels drastically narrows the electron density contrast between the COF skeleton and the pore interior. This structure-factor redistribution is further verified by the concurrent intensity enhancements in the (210), (220), and (330) reflections, confirming that the parent crystalline lattice remains strictly intact. In the out-of-plane direction, the (001) reflection experiences a minor shift to *Q*_*z*_ = 1.57 Å^–1^, corresponding to a slightly expanded interlayer *d*-spacing of ∼ 4.0 Å. This subtle dilation along the stacking direction is perfectly consistent with the added steric bulk of the naphthyl groups pushing the 2D sheets slightly apart (Figures S7c,d).

**Figure 2:**
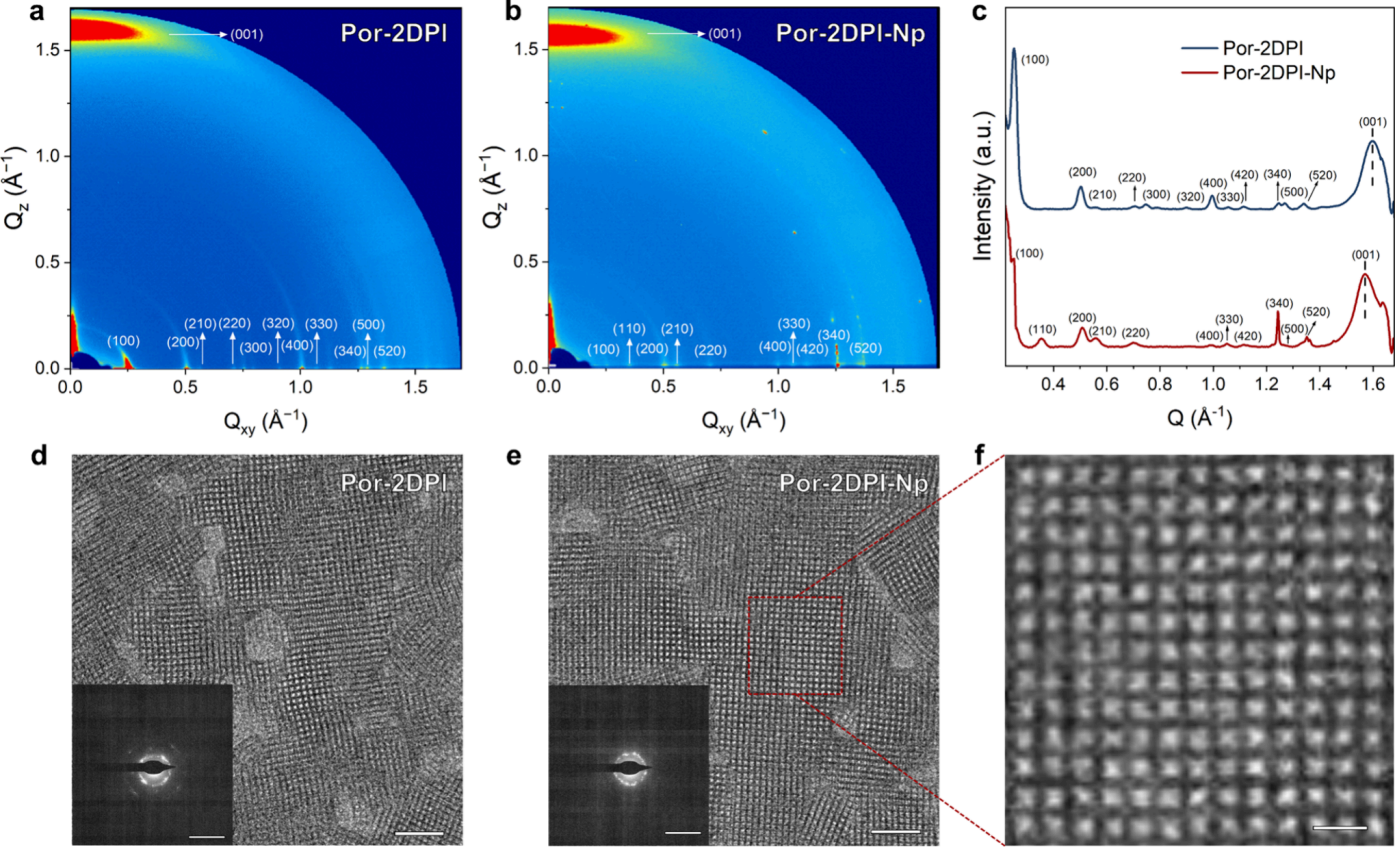
(a and b) GIWAXS images of Por-2DPI and Por-2DPI-Np COF membranes, respectively. (c) Projection of GIWAXS data from (a) and (b). (d and e) HRTEM images of Por-2DPI and Por-2DPI-Np. (f) Enlarged HRTEM image showing the ordered square lattice in Por-2DPI-Np. Scale bars: 20 nm in (d) and (e), 1 nm^–1^ in the inset images of (d) and (e), 5 nm in (f).

To quantify their mechanical properties, the intact Por-2DPI, Por-2DPI-Ph and Por-2DPI-Np membranes were assembled into ∼ 32 nm thick films (corresponding to the optimized thickness for gas separation, as discussed later) and transferred onto bare copper grids, where they were seamlessly suspended over the microholes (∼20 μm in diameter) for nanoindentation measurements (Figure S8). All evaluated membranes exhibited robust elastic moduli consistently exceeding 30 GPa. These results confirm that regardless of the specific inner-pore ligands, the ultrathin continuous frameworks possess sufficient mechanical robustness to withstand practical handling and pressurized gas separation.

We evaluated the local crystallinity and molecular alignment of these films using high-resolution transmission electron microscopy (HRTEM) and selected area electron diffraction (SAED). For the pristine Por-2DPI, HRTEM imaging (Figure 2d) resolves highly ordered square lattices within local crystalline domains, exhibiting an interplanar spacing of 2.50 nm. The corresponding SAED pattern (Figure 2d inset) presents distinctive diffraction rings comprising discernible spots at 0.4 nm^–1^. This perfectly corroborates the GIWAXS results, indicating that while the membrane is macroscopically polycrystalline with visible grain boundaries (Figure S9), it is assembled from numerous highly ordered, single-crystalline domains. The distinct ring pattern confirms that these domains maintain a strict face-on orientation, providing vertically aligned 1D channels across the film at the micrometer scale (Figure S10). Despite the dense grafting of bulky phenyl or naphthyl groups within their pores, the targeted Por-2DPI-Ph (Figure S11) and Por-2DPI-Np (Figure 2e,f) membranes strictly preserve this structural order. HRTEM imaging of Por-2DPI-Np continues to display clear, ordered arrays of square porous channels within its local domains. Alongside this, its SAED pattern (Figure 2e inset) completely retains the characteristic diffraction rings and the associated subtle spots. This demonstrates that the in situ IPC strategy successfully constricts the pore environment without disrupting the underlying crystalline framework or the critical face-on alignment.

### Porosity Analysis of 2DPI

To investigate how the IPC strategy influences the porous properties of these 2D COF membranes, we first performed comparative pore analysis on their corresponding bulk powder samples. The 2DPI powder samples were synthesized via solvothermal methods, and the Por-2DPI-R (R = Ph and Np) powders were subsequently prepared via the developed IPC strategy (synthesis details shown in SI). The powder XRD (PXRD) measurements (Figure 3a) demonstrated the high crystallinity of the achieved Por-2DPI and Por-2DPI-R powders, which present the major peaks at 3.5°, 7.1° and 21.7°, assigned to (100), (200), and (001) planes, respectively. In this case, we analyzed the porosity of these COF powders via N_2_ adsorption isotherms tests at 77 K (Figure 3b). The results revealed a Brunauer–Emmett–Teller (BET) surface area of 1144 m^2^ g^–1^ for the pristine Por-2DPI powder, whereas the corresponding BET values for Por-2DPI-Ph and Por-2DPI-Np were significantly reduced to 873 m^2^ g^–1^ and 334 m^2^ g^–1^, respectively. The pore size distribution obtained through the NLDFT model (Figure 3c) also suggested a decrease of the pore sizes from 2.2 nm (Por-2DPI) to 1.7 nm (Por-2DPI-Ph) and 1.3 nm (Por-2DPI-Np) due to constriction of the porous channels by the grafted aryl groups.

**Figure 3:**
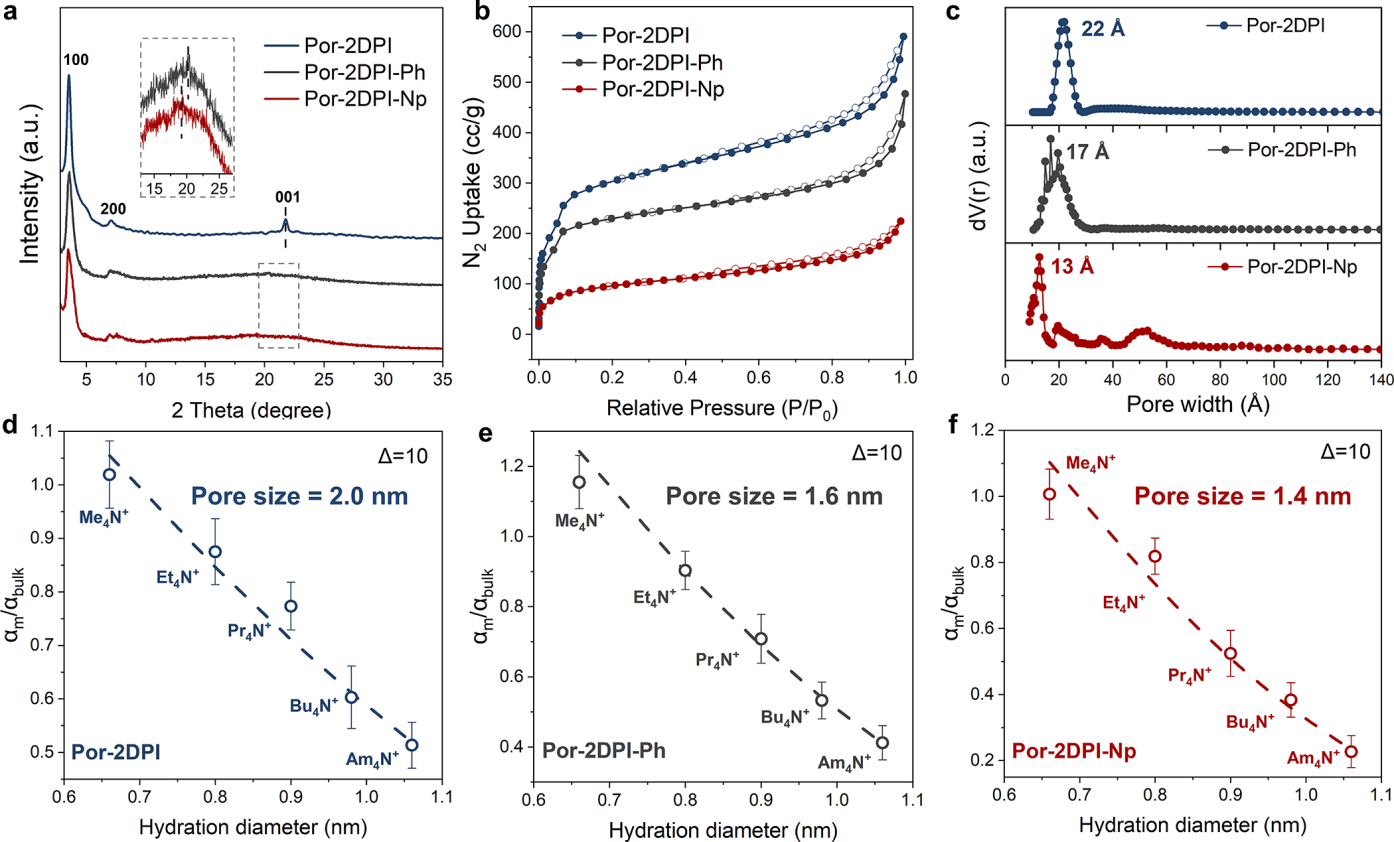
(a) PXRD patterns of the resultant 2D COF powder. (b) N_2_ adsorption–desorption isotherms. (c) Pore size distribution calculated by the nonlocal density functional theory (NLDFT) model. (d–f) Relative mobility ratios of different tetraalkylammonium cations to Cl^–^ (normalized by their mobilities in bulk solutions) for Por-2DPI (d), Por-2DPI-Ph (e) and Por-2DPI-Np (f) membranes. The dashed line is the best fit to the data using a hydrodynamic model.

Pore size estimation of 2DPI membrane samples was further conducted via ionic conductance measurements using tetraalkylammonium chlorides [(C_n_H_2n+1_)_4_N]Cl with alkyl chain lengths n = 1 ∼ 5. These ions exhibit minimal membrane interaction changes with size, allowing pore size assessment based on steric hindrance. Normalized cation mobility (α_m_/α_bulk_) decreases with increasing ion size, following the relation:^31,32^

$$\frac{\alpha_{m}}{\alpha_{bulk}}=c{\left[1-\frac{d}{D}\right]}^{2}$$

where *d* is the hydrated ion diameter, *D* is the pore diameter, and *c* is a constant (the calculation details are provided in the Supporting Information). Figures 3d–f indicate that the pore size of Por-2DPI membrane was calculated to be ∼ 2.0 nm using the above formula, while the pore sizes of Por-2DPI-Ph and Por-2DPI-Np membranes were determined as ∼ 1.6 nm and ∼ 1.4 nm, respectively. The porosity analysis results for both powder and membrane samples are in excellent agreement, further demonstrating the success of the IPC strategy in efficiently tailoring the internal nanochannels; the effective pore size systematically decreases as the steric bulk of the grafted aryl groups increases.

### Gas Separation Performance

The as-prepared Por-2DPI and Por-2DPI-R membranes were transferred onto PAN substrates for He/CH_4_ and He/N_2_ separation tests (Figures 4a and S12). Single-gas permeances were evaluated using a constant-volume/variable-pressure permeation system (Figure S13) at 25 °C and 1 bar. Following overnight degassing in the sample cell, steady-state pressure increments were recorded upon the introduction of various feed gases (He, H_2_, CO_2_, N_2_, and CH_4_) under controlled upstream pressures. To ensure measurement precision for these high-permeance membranes, the effective testing areas were precisely calibrated via optical microscopy (Figure S14). To investigate the influence of membrane thickness on separation performance, two approaches were employed: in situ synthesis of membranes with variable thicknesses (Figure 1d) and controlling the number of transfer cycles (Figures 4a, S15, and S16). First, to decouple intrinsic transport properties from macroscopic thickness, a series of Por-2DPI membranes (4 to 200 nm thick) were prepared by adjusting monomer concentrations (Figure 1d). As shown in Figure S17, the He/CH_4_ selectivities remained low (1.6–3.1) across all thicknesses, aligning closely with the theoretical Knudsen diffusion selectivity (∼2.0) for this gas pair. This indicates that gas transport in these unstacked membranes is governed primarily by relative molecular velocities rather than size-exclusion.

**Figure 4:**
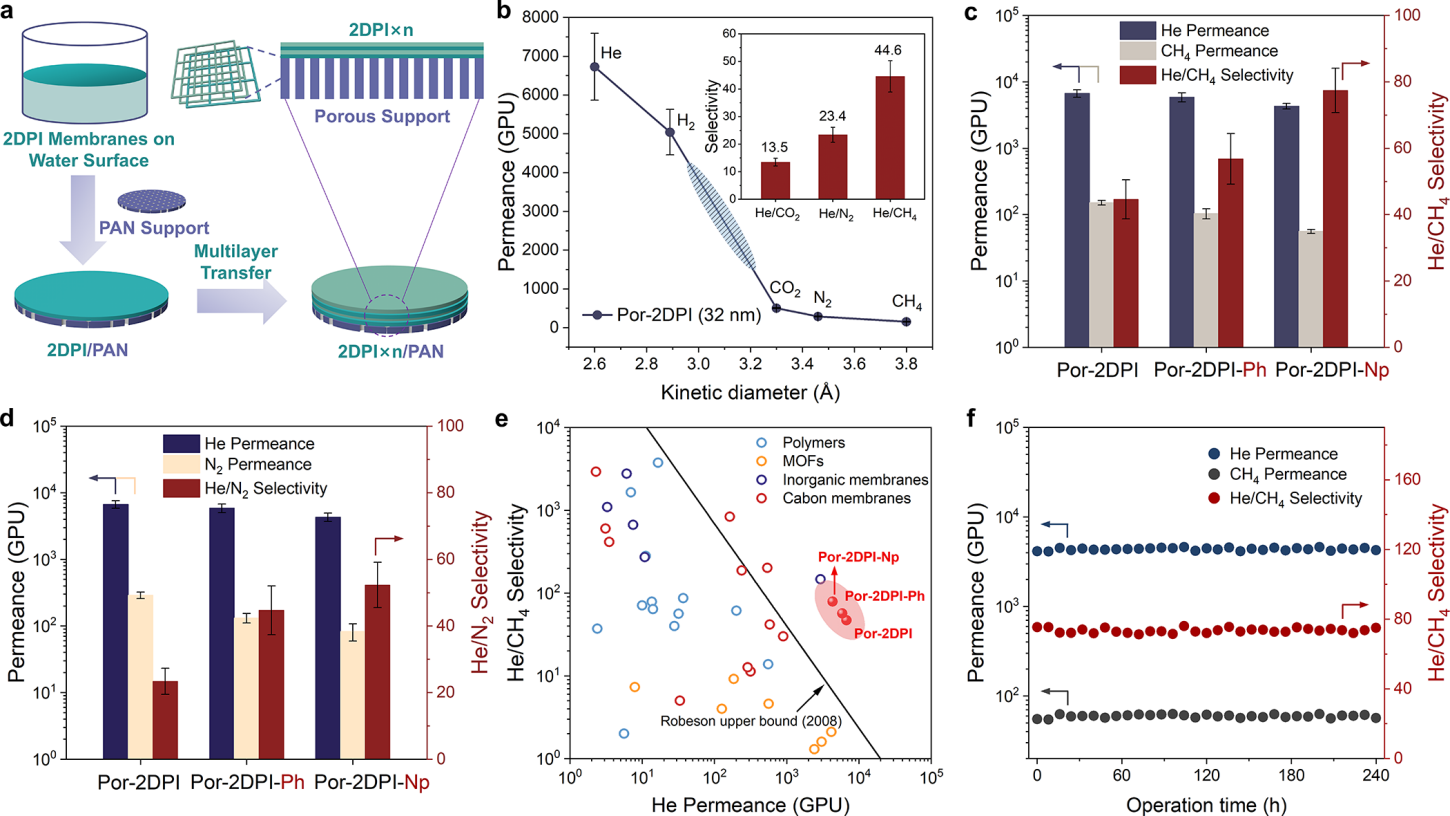
(a) Scheme of the layer-by-layer transfer of 2DPI COF membranes onto the PAN substrate. (b) Single-gas permeance of a 32 nm-thick Por-2DPI membrane versus kinetic diameter of gas molecules. (Inset: the corresponding ideal selectivities for relevant gas pairs.) (c) He/CH_4_ separation performance of developed COF membranes. (d) He/N_2_ separation performance of developed COF membranes. (e) The performance comparison of He/CH_4_ separation between the prepared COF membranes with the other reported membranes. (f) Long-term He/CH_4_ mixed-gas separation stability of the Por-2DPI-Np membrane.

Subsequently, we examined the performance of COF membranes assembled via repeated transfers (Figure S18). When evaluating membranes prepared with 1, 3, and 6 transfer cycles (corresponding to thicknesses of 4, 18, and 32 nm, respectively; Figure S15), the He permeance systematically decreased from 39262 GPU to 6727 GPU. The microstructural characterizations confirm that this alternating layer-by-layer assembly, coupled with mild thermal annealing, ensures seamless interlayer adhesion, thereby yielding a highly integrated macroscopic architecture (Figures S15, S16, and S19). Concurrently, the He/CH_4_ selectivity exhibited a remarkable enhancement from 1.2 to 44.6. The high He permeance is sustained by the ultrathin profile (<50 nm) and continuous 1D channels, whereas the pronounced improvement in selectivity is attributed to the dense, statistical random overlapping of the intrinsic 2D pores between adjacent layers across the transmembrane pathway. Further increasing the thickness to 48 nm (9 transfer cycles) yielded a marginal selectivity increase to 48.5, but at the cost of a significant permeance reduction (retaining only 44% of the flux observed for the 32 nm membrane), indicating that the statistically driven pore-narrowing effect has already reached its functional limit at 6 transfer cycles (Figure S20). Therefore, the 6-cycle (∼32 nm) architecture was identified as the optimal platform for subsequent structural engineering and separation evaluations.

The gas separation performance of the optimized 6-cycle Por-2DPI membranes (transferred 6 times, ∼ 32 nm, Figures S15 and S16) was systematically evaluated across various gas probes. As shown in Figure 4b, the single-gas permeance exhibits a strong dependence on the kinetic diameter of the penetrant molecules, which is highly consistent with the size-sieving mechanism discussed above. The pristine Por-2DPI membrane delivered exceptionally high permeances for small gases (<3 Å), reaching 6727 GPU for He and 5041 GPU for H_2_. However, a drastic decline in permeance occurs for larger molecules. The permeance drops to 500 GPU for CO_2_ (3.3 Å) and 287 GPU for N_2_ (3.64 Å), before further declining to 151 GPU for CH_4_ (3.8 Å). This sharp size-exclusion cutoff, precisely located between H_2_ (2.89 Å) and CO_2_ (3.30 Å), empirically confirms that the effective transport bottlenecks—created by the dense turbostratic overlapping of intrinsic pores across multiple atomic layers—are strictly constricted to the 2.9–3.3 Å regime, perfectly matching the requirements for He/CH_4_ sieving. Driven by this clear kinetic cutoff, the membrane yields ideal selectivities of 13.5 for He/CO_2_ and 23.4 for He/N_2_. Notably, the He/CH_4_ selectivity reached 44.6 (Figure 4b inset), already exceeding the performance of typical commercial polymer membranes (20–30).^1^ Crucially, while this staggered stacking establishes the fundamental dimensional threshold (providing a baseline sieving effect), the local chemical environment within these channels can be further engineered to amplify the sieving precision, a concept we subsequently validate via the IPC strategy.

Subsequent IPC modification further refined the pore channels to intensify the steric hindrance against larger gas (Figure S21). While the He permeance of the modified COF membranes (Por-2DPI-Ph and Por-2DPI-Np) decreased to 5902 GPU and 4323 GPU, their He/CH_4_ selectivities were significantly enhanced to 56.8 and 77.4, respectively (Figure 4c). The He/N_2_ separation performance followed a similar improving trend (Figure 4d), with the selectivity systematically increasing from 23.4 for the pristine membrane to 44.7 and 52.3 for Por-2DPI-Ph and Por-2DPI-Np, respectively. This combination of high He permeance and exceptional selectivity for both gas pairs (He/CH_4_ and He/N_2_) places the performance of our membranes, particularly Por-2DPI-Np, well above the 2008 Robeson upper bounds (Figure 4e, Figure S22, and Table S1).^9^ Notably, the separation performance remained consistent across five independent batches and scaled-up testing areas (RSD < 10%), confirming the scalability and reproducibility of the IPC strategy (detailed in Table S2 and Figure S23). To elucidate the mass transport mechanism, temperature-dependent permeation was evaluated for both the pristine and modified membranes (Figures S24 and S25). Compared with the pristine membrane (where *E*_a_ values for He and CH_4_ were 2.9 and 8.7 kJ mol^–1^, respectively), the derived single-gas activation energies (*E*_a_) of the Por-2DPI-Np membrane follow a strict size-dependent trend: He (4.4 kJ mol^–1^) < CO_2_ (6.5 kJ mol^–1^) < N_2_ (9.9 kJ mol^–1^) < CH_4_ (13.1 kJ mol^–1^). The substantial escalation of the CH_4_ transport barrier (from 8.7 to 13.1 kJ mol^–1^) after IPC modification provides direct kinetic proof for a rigid size-sieving mechanism induced by engineered pore narrowing. Practical separation capability was evaluated using an equimolar He/CH_4_ mixture (Table S3). Temperature-dependent analyses (Figure S26) show that while competitive hindrance slightly raises the *E*_a_ of He to 5.6 kJ mol^–1^, the *E*_a_ of CH_4_ remains dictated by rigid steric confinement (13.2 kJ mol^–1^). Preserving this large kinetic gap confirms the robustness of the size-sieving effect during competitive transport. Furthermore, a 240-h mixed-gas (equimolar He/CH_4_) test (Figure 4f) confirmed the stable operation of the Por-2DPI-Np membrane, indicating its strong potential for industrial application. Post-test characterizations (Figure S27) revealed no discernible structural degradation, indicating that the intrinsic chemical bonds and staggered LbL architecture effectively withstand prolonged transmembrane pressure. Remarkably, the membrane also maintained fully reversible separation performance under demanding thermal cycling (10 to 55 °C) and dynamic pressure shocks (1 to 4 bar) (Figures S24 and S28).

### Mechanistic Understanding of He Separation

The exceptionally high gas permeance of membranes in this study is primarily attributed to their ultrathin nature and the continuous 1D porous channels resulting from high crystallinity. To understand the mechanism behind the enhanced selectivity, we deconvoluted the individual contributions of the modified pores and the macroscopic stacking. We first tested 4 nm single-cycle Por-2DPI-Ph and Por-2DPI-Np films (Figure S29), which exhibited only Knudsen-like He/CH_4_ selectivity. This confirms that intrinsic pore constriction alone is insufficient for effective molecular sieving over such a short diffusion distance. Therefore, the high selectivity of the assembled membranes must originate from a structural-chemical synergy. Specifically, the multicycle transfer induces turbostratic stacking, constructing tortuous, subnanometer pathways that provide a basic dimensional threshold for size-exclusion. Within these structurally confined channels, the grafted aryl groups act as localized steric hurdles to further impede the diffusion of larger gas molecules, kinetically amplifying the overall separation performance (Figure 5a). Pore size distribution analysis (Figures 5b and S30) confirms that this combination significantly narrows the effective transport channels. The kinetic nature of this molecular sieving mechanism is corroborated by both the sharp size-exclusion cutoff in Figure 4b and the negligible time-lag recorded during permeation measurements. The lack of a discernible delay explicitly rules out the dissolution-diffusion pathways typical of polymeric membranes, confirming that CH_4_ transport is instead restricted by dynamic physical collisions within the confined channels. Molecular dynamics (MD) simulations of equimolar He/CH_4_ mixtures were performed to provide clear mechanistic insight into this strategy’s effectiveness (Figures 5c,d and S31). The comparative multilayer MD models employ the intrinsic crystallographic interlayer spacings (0.39 and 0.40 nm) derived from GIWAXS, rather than the macroscopic envelope thickness measured by AFM. These tightly bound intrinsic contact domains accurately capture the microscopic rate-determining bottlenecks where the actual size-exclusion occurs. After 200 ps of simulation, the number of permeating He molecules in the IPC-modified Por-2DPI-Np membrane decreased by 14% compared to the pristine membrane. In stark contrast, the permeation of larger CH_4_ molecules was heavily suppressed, showing a significant 67% reduction (Figure 5c). Consequently, as shown in Figure S32, the separation factor increased from 4.9 (pristine) to 12.6 (modified). This asymmetric mass transport is the direct cause of the enhanced separation factor. The results clearly demonstrate that the bulky aryl groups grafted via IPC introduce severe spatial hindrance within the pores, thereby imposing a substantially higher diffusion barrier for the larger CH_4_ molecules compared to the ultrasmall He atoms (Figure 5a). Given the highly similar kinetic diameters of N_2_ (3.64 Å) and CH_4_ (3.8 Å), this strongly constricted pore environment imposes a comparably prohibitive diffusion energy barrier for N_2_ molecules. This effectively arrests the transport of these large penetrants while allowing the ultrasmall He atoms to pass with minimal resistance. This selective restriction is the key mechanism by which the IPC strategy enhances molecular sieving selectivity without severely compromising the high helium permeance.

**Figure 5:**
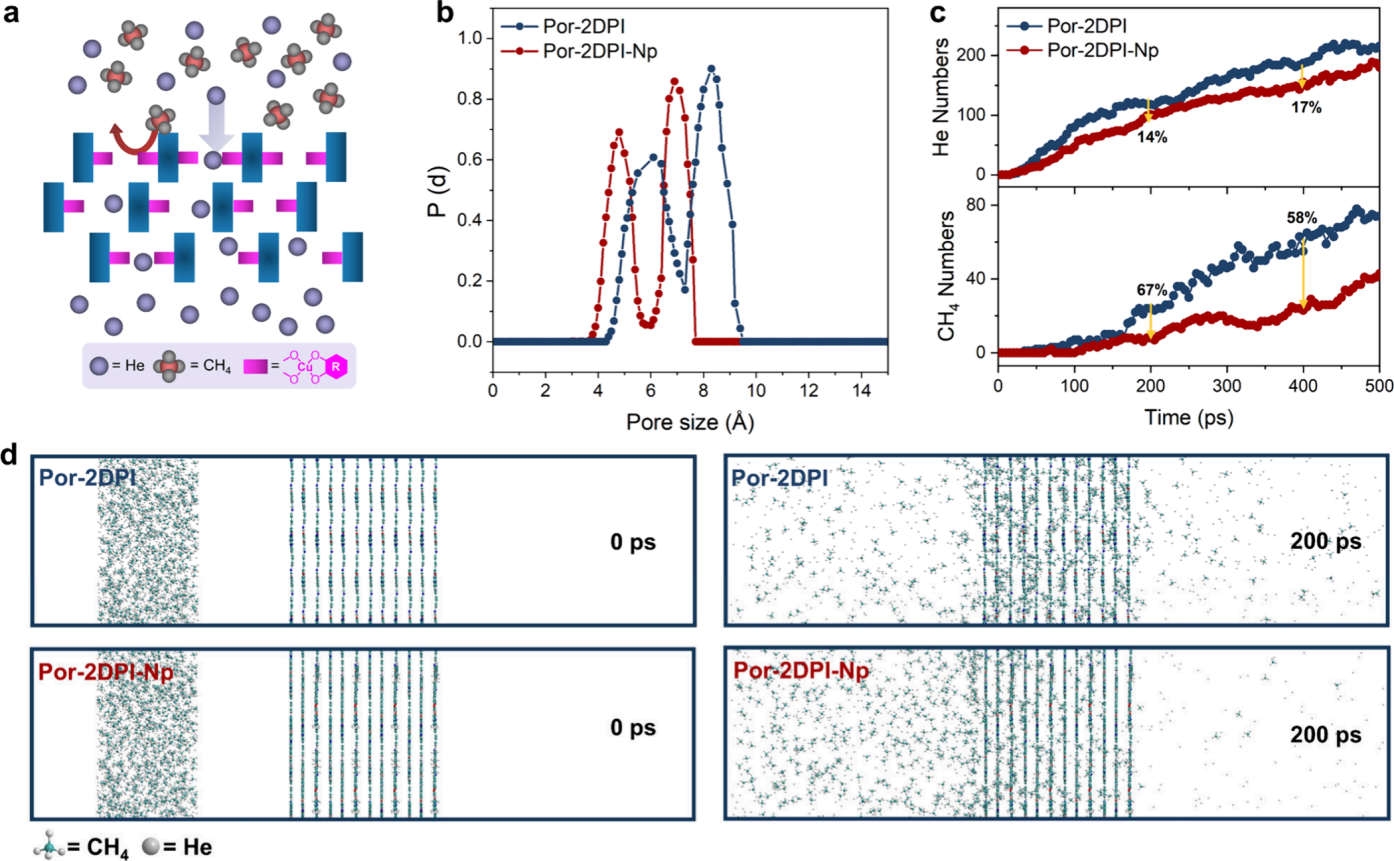
(a) Schematic diagram of gas molecules passing through Por-2DPI-R. (b) Pore size distribution of Por-2DPI and Por-2DPI-Np of stacked six layers obtained from simulated 2DP models. (c) The permeation numbers of gas molecules through membranes within 500 ps in molecular dynamics simulations. (d) Simulation system with snapshot at 0 ps, 200 ps for the permeation of equimolar helium and methane through Por-2DPI and Por-2DPI-Np.

## Conclusions

In summary, this work demonstrates a powerful pore-engineering approach for fabricating ultrahigh-performance helium separation membranes based on fully crystalline 2DPI COFs. The core of our strategy lies in the IPC, which enables the precise chemical tailoring of the 1D porous channels. By grafting aryl groups of different sizes (phenyl and naphthyl) onto the coordinative sites within the pores, we achieved effective pore constriction, creating a tailored sieving environment that preferentially impedes the diffusion of larger CH_4_ molecules over He. The exceptional separation performance stems from the synergistic integration of three key structural factors: (i) ultrathin (∼32 nm), face-on oriented membrane that minimizes the diffusion path length; (ii) the well-defined 1D porous channels providing straight pathways for rapid gas transport; and (iii) the IPC-based pore engineering that precisely modulates the channel size and chemistry. This synergy allows the optimized Por-2DPI-Np membrane to simultaneously achieve an ultrahigh He permeance exceeding 4300 GPU and an exceptional He/CH_4_ selectivity of 77.4, placing its performance above the current state-of-the-art upper bound. Our findings highlight the critical role of atomic-scale channel design in overcoming the pervasive permeability–selectivity trade-off. The IPC strategy presented here is not limited to He separation but offers a versatile and generalizable route to engineer advanced porous membranes for other challenging molecular separations, such as H_2_/CH_4_ or CO_2_/N_2_, by simply varying the coordinating ligands. It should be noted that while precise gas sieving inherently depends on strict geometric matching—for instance, parent pores larger than 3.0 nm remain too wide for gas separation even after IPC modification—the broad chemical versatility of this strategy opens new avenues beyond gas transport. By accommodating diverse COF skeletons and coordinating ligands, this approach shows potential liquid-phase applications, including organic solvent nanofiltration and precise ion separation. This work enhances the fundamental understanding of molecular transport in 2D COF membranes and provides a blueprint for developing highly selective, high-flux He membranes through atomic-scale design.

## Supplementary Material



## Notes

Experimental methods, synthetic procedures, characterization data, AFM and SEM images, XPS spectra, nanoindentation measurements, gas permeation performance, and molecular dynamics (MD) simulation details (PDF)
