# Supplementary material for: Ultrathin Covalent Organic Framework Membranes with Tailorable Porous Channels for High-Permeance Helium Separation
Source: J Am Chem Soc. 2026 Jun 4;148(23):24096–105. doi: 10.1021/jacs.6c04426 (PMC13281534; doi:10.1021/jacs.6c04426)
Supplement: Supplementary file 1 [file ja6c04426_si_001.pdf]

Ultra-Thin Covalent Organic Framework Membranes with Tailorable Porous Channels for High-Permeance Helium Separation

*Wei Xie,[1,2,3] Fengxiang Zhao,[2,3] Tengyang Zhu,[1,2,3] Chao Sun,[2,3] Jichao Zhang[4], Renhao Dong [2,3,]**

[1]Key Laboratory of Colloid and Interface Chemistry, School of Chemistry and Chemical Engineering, Shandong University, Jinan, 250100, China

[2]Department of Chemistry, The University of Hong Kong, Hong Kong, 999077, China

[3]Materials Innovation Institute for Life Sciences and Energy (MILES), HKU-SIRI, Shenzhen 518048, China

[4]Shanghai Synchrotron Radiation Facility, Shanghai Advanced Research Institute, Chinese Academy of Sciences, Shanghai 201204, China

Materials

4,4',4'',4'''-(21H,23H-porphyrin-5,10,15,20-tetryl)tetraaniline (TAPP), 2,3-dihydroxybenzene-1,4-dicarbaldehyde (2,3-DHTA), catechol and 2,3-dihydroxynaphthalene were obtained from Aladdin Chemicals (Beijing) and used directly. Trifluoromethanesulfonic acid was purchased from Sigma-Aldrich. Other chemicals were purchased and used without further treatment. The polyacrylonitrile (PAN) ultrafiltration membranes were commercially supplied by RisingSun Membrane Technology (Beijing) Co., Ltd. The chosen PAN support features a nominal molecular weight cut-off (MWCO) of approximately 50000 Da, which possesses an appropriately smooth surface and uniform nanopores (~10 nm).

Characterizations

Optical microscopy (OM) images were recorded on Leica DM2000 LED microscope. Attenuated total reflection infrared spectroscopy (ATR-IR) was measured by Fourier infrared spectroscopy (Tensor II) with an instrument resolution of 4 cm^{-1} and a scanning range of 4000-600 cm^{-1}. Raman spectroscopy was performed by in situ ultraviolet confocal Raman spectrometer (LabRAM HR Evolution) with spectral range of 200 nm-1100 nm. Spectral resolution: infrared ≤ 0.35 cm^{-1}; Visible ≤0.65 cm^{-1}; ultraviolet ≤ 1.6 cm^{-1}. X-ray photoelectron spectroscopy (XPS) was performed using a Thermo Fisher ESCALAB XI+ spectrometer. The X-ray source is AlKα (1486.71 eV), and the sample is loaded onto a silicon substrate prior to the test. The membrane thickness was measured by atomic force microscopy (AFM) performed on a Bruker BioScope Resolve instrument (peak force tapping mode). HRTEM images were recorded by a Thermo Fisher Scientific-Talos F200S operated with the acceleration voltage of 200 kV at room temperature. Powder X-ray diffraction (PXRD) patterns were recorded on a Smart Lab 9 KW for Cu Ka radiation (λ = 1.5406 Å), with a scan speed of 10° min^{-1}. The crystal orientation of two-dimensional polymers was measured by grazing incidence wide-angle X-ray scattering (GIWAXS). The experiments were carried out on the BL15U1 beamline of Shanghai Synchrotron Radiation Facility (SSRF, China). The detector is a Mar165 charge-coupled device (CCD), the photon beam energy is 12.398 keV (λ= 1Å), the distance from the sample to the detector is 301.121 mm, and

the detector is calibrated with lanthanum hexaboride (LaB_6). The grazing incidence angle is 0.15° and the exposure time is 20 s. The Dioptas software was used to analyze the data. The nanoindentation measurements were explicitly conducted on the freestanding (suspended) membrane regions spanning the holes of a bare copper grid using a KLA G200 Nanoindenter. The membranes tested herein were prepared with 6 transfer cycles, corresponding to a precisely controlled thickness of ~32 nm. To ensure statistical reliability, the indentations were performed at 3 to 5 randomly selected suspended regions, and the results were averaged.

Synthesis of Por-2DPI Powder

TAPP (0.02 mmol, 13.48 mg) and 2,5-DHTA (0.04 mmol, 6.64 mg) were added to an o-DCB/BuOH/6 M AcOH mixed solution (5/5/1 by vol.; 1.1 mL) in a Pyrex tube (10 mL). After three freeze-pump-thaw cycles, the tube was heated at 120 °C for three days. The precipitate was washed with anhydrous THF and acetone. The powder was dried at 120°C under vacuum overnight to give the product in a yield of 78%.

Synthesis of Por-2DPI-R Powder

A certain amount of Por-2DPI (10.00 mg) powder and $Cu(OAc)_2 \cdot H_2O$ (0.2 g, 1.124 mmol) were mixed in a methanol/water mixed solution (20 mL, v/v = 1:1) and stirred at 80°C for 12 h. Then monomer 3 (catechol or 2,3-dihydroxynaphthalene, 2.25 mmol) was added to the mixture and the reaction continued for 24 h. After centrifugation, the product was washed with water and methanol then dried under vacuum, and its yield was 85%.

Macroscopic Membrane Transfer Procedure

The transfer of the Por-2DPI membranes onto solid substrates (e.g., Si/SiO_2, copper foil, and PAN) was performed using a horizontal transfer technique.

Horizontal transfer: The target substrate was first wetted and submerged beneath the air–water interface. It was then horizontally positioned to contact the floating COF film and gradually withdrawn, allowing the film to deposit flatly onto the substrate without severe wrinkling.

Drying and adhesion: To remove interfacial water and promote physical adhesion between the COF film and the substrate (or between adjacent domains during multi-

cycle assembly), the newly transferred sample was placed in a forced-air oven at 60 °C for 5 min, followed by vacuum drying for 30 min. This mild thermal treatment assists in flattening the membrane surface. The dried membrane was subsequently rinsed with trace amounts of ethanol and deionized water.

Multi-cycle assembly: For the preparation of thicker membranes (e.g., 3, 6, and 9 transfer cycles), the above-mentioned "transfer–drying–washing" protocol was repeated iteratively.

Gas Permeation Measurements

Pure gas permeance measurements were conducted with five gases (i.e., He, H_2, CH_4, N_2, and CO_2) at 25 °C and 1 bar (1 bar = 100 kPa) using a custom-designed permeation system according to the constant-volume/variable-pressure method. The downstream and upstream chambers in the testing equipment were degassed for at least 8 h before measurements. The upstream pressure was held at predetermined pressures, and the steady-state pressure increase rate was monitored by the downstream transducer. The permeance (P) was determined as follows

$$P = 10^6 \frac{T_0 V_d}{ATP_0 \Delta p}\left[\left(\frac{dp}{dt}\right)_{ss} - \left(\frac{dp}{dt}\right)_{leak}\right]$$

where: P (GPU, 1 GPU = 10^{-6} cm^3 (STP) cm^{-2} s^{-1} $cmHg^{-1}$) is the gas permeance. V_d is the calibrated downstream volume (38 cm^3). T_0 is the standard temperature (273.15 K). P_0 is the standard pressure (76 cmHg). A is the effective membrane testing area (cm^2). To accommodate the exceptionally high permeance within the fixed downstream volume, the effective area was restricted using an impermeable epoxy/aluminum mask to prevent rapid transducer saturation and ensure a highly linear pressure accumulation. T is the absolute test temperature (298.15 K). Δp is the transmembrane pressure difference (cmHg). Prior to testing, the downstream chamber was thoroughly degassed to a vacuum state (< 50 Pa), making the downstream pressure completely negligible compared to the upstream pressure. $\left(\frac{dp}{dt}\right)_{ss}$ is the downstream steady-state pressure increment rate (cmHg/s). $\left(\frac{dp}{dt}\right)_{leak}$ is the system leak rate (cmHg/s). Note: The background leak rate of our permeation system was strictly below

2×10^{-7} cmHg/s, which corresponds to less than 1% of the downstream steady-state pressure increment for all tested gases. This minimal leakage was rigorously subtracted during the calculation to ensure accuracy. All reported gas permeance and selectivity values are averages, and the corresponding error bars represent the standard deviations calculated from 3 to 5 independently fabricated membrane samples.

The ideal gas selectivity of two gases of A and B was defined as the pure gas permeance ratio, with A being the more permeable gas: $\alpha_{A/B} = P_A/P_B$.

The mixed-gas permeation was measured at 25 °C using a custom-designed constant volume/variable pressure apparatus. A binary He/CH_4 gas mixture of 50:50 was used, and the total feed pressure was 1 bar, and the downstream pressure was less than 0.01 bar. The stage-cut was less than 0.5% to ensure that the residue composition was essentially unchanged. The composition of the binary permeate gas mixture was determined by a gas chromatograph (Shimadzu GC-2014 system) equipped with thermal conductivity detectors and a flame ionization detector. The mixed-gas permeance of gas A was determined as follows

$$P_A = 10^6 \frac{y_A V_d T_0}{x_A \Delta p A T P_0} \frac{dp}{dt}$$

where P_A is the mixed-gas permeance of component A (GPU, 1 GPU = 10^{-6} cm^3 (STP) cm^{-2} s^{-1} $cmHg^{-1}$), and y_A and x_A are the mole fractions of component A in the permeate and feed streams, respectively. The mixed-gas selectivities were obtained by:

$$\alpha_{A/B} = \frac{y_A/y_B}{x_A/x_B}$$

Calculation of the Effective Pore Size

The effective sub-nanometer pore sizes (D) of the membranes were evaluated based on the hindered diffusion of organic cations. A series of tetraalkylammonium chloride solutions with a concentration gradient (Δ = 10) were employed as the testing electrolytes, including tetramethylammonium (Me_4N^+, d = 0.66 nm), tetraethylammonium (Et_4N^+, d = 0.80 nm), tetrapropylammonium (Pr_4N^+, d = 0.90 nm), tetrabutylammonium (Bu_4N^+, d = 0.98 nm), and tetrapentylammonium (Am_4N^+, d = 1.06 nm).

The relative cross-membrane ion mobility ratio (α_m) of the specific cation relative

to Cl⁻ was directly extracted from the experimentally measured open-circuit potential (V_0) using the Henderson equation[1, 2]:

$$\alpha_m = \frac{\mu_+}{\mu_{Cl}} = -\frac{z_+}{z_-} \cdot \frac{ln\,(\Delta) - \frac{z_- F V_0}{RT}}{ln\,(\Delta) - \frac{z_+ F V_0}{RT}}$$

where $\mu_+$and μ_{Cl} are the ionic mobilities of the respective cations and Cl⁻; z_+ and z_- are the charge valences of the cations and anions (Cl⁻); F is the Faraday constant; R is the universal gas constant, and T is the experimental temperature (298.15 K).

Subsequently, the relationship between the effective membrane pore size (D) and the ion diameter (d) was fitted using the hydrodynamic hindered diffusion model [Ref]:

$$\frac{\alpha_m}{\alpha_{bulk}} = c[1 - \frac{d}{D}]^2$$

where α_{bulk} is the inherent bulk mobility ratio of the specific cation to Cl⁻ in a free solution, and c is a constant coefficient related to the pore geometry. By plotting the experimentally derived relative mobility ratio ($\frac{\alpha_m}{\alpha_{bulk}}$) against the hydrated diameters (d) of the testing cations, the effective pore diameter (D) was precisely extracted via non-linear curve fitting.

Simulation Details and Methodology

The atomistic force field parameters are described by the OPLS-AA force field.[3] The partial charges used were 1.2×CM5.

Membrane Model Construction: To accurately simulate the physical environment of transmembrane gas permeation and decouple the separation mechanism, two comparative gas separation models (the pristine Por-2DPI and the modified Por-2DPI-Np) were constructed. For each system, the size of the simulation box was set to x = 15.0 nm, y = 15.0 nm, and z = 40.0 nm. The box is divided into three regions by the Por-2DPI membrane placed in the middle. The left region (feed side) was randomly inserted with 1000 He and 1000 CH_4 molecules, while the right region served as the permeate side.

Crucially, the membrane model was constructed using a 6-layer turbostratic

(staggered) stacking configuration. It should be noted that while the macroscopic apparent thickness of the multi-layer transferred films (measured via AFM) suggests a larger average gap (>1.6 nm) due to transfer-induced mesoscopic wrinkles and interfacial voids, the actual molecular sieving strictly occurs at the local microscopic domains where adjacent COF layers are in tight physical contact. Because penetrating these tightly stacked π-π domains is the rate-determining bottleneck for gas transport, the interlayer spacings in our MD models were explicitly set to match their respective intrinsic GIWAXS data: 0.39 nm for the pristine Por-2DPI model and 0.40 nm for the modified Por-2DPI-Np model. Simulating these tight intrinsic spacings, coupled with random in-plane offsets, rigorously captures the true steric bottlenecks. By comparing these two models, we can intuitively visualize how the grafted -Np groups act as localized steric hurdles to amplify the baseline size-exclusion effect.

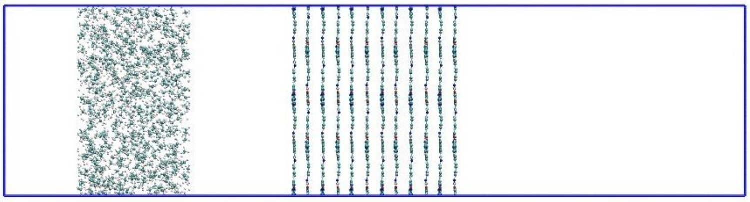

Simulation Parameters: The molecular force field consists of nonbonded and bonded interaction. The nonbonded interaction contains van der Waals (vdW) and electrostatic interaction, which is described by these equations.

$$E_{LJ}(r_{ij}) = 4\varepsilon_{ij}\left(\left(\frac{\sigma_{ij}}{r_{ij}}\right)^{12} - \left(\frac{\sigma_{ij}}{r_{ij}}\right)^{6}\right)$$

$$E_c\left(r_{ij}\right) = \frac{q_i q_j}{4\pi\varepsilon_o\varepsilon_r r_{ij}}$$

In the equation, q_i, q_j are atomic charge, r_{ij} is the distance between atoms, σ is the atomic diameter, ε is the atomic energy parameter.

For different kinds of atoms, the Lorentz-Berthelot rules were adopted for vdW interactions, which is following equation. The cutoff distance of vdW and electronic interactions was set to 2.0 nm, and the PME method[4] was employed to calculate long-range electrostatic interactions.

$$\sigma_{ij} = \frac{1}{2}(\sigma_{ii} + \sigma_{jj}); \varepsilon_{ij} = (\varepsilon_{ii} \times \varepsilon_{jj})^{\frac{1}{2}}$$

Atomistic simulations were performed using GROMACS package with cubic periodic boundary conditions. The equations for the motion of all atoms were integrated using a classic Verlet leapfrog integration algorithm with a time step of 1.0 fs. A cutoff radius of 2.0 nm was set for short-range van der Waals interactions and real-space electrostatic interactions. The particle-mesh Ewald (PME) summation method with an interpolation order of 4 and a Fourier grid spacing of 0.16 nm was employed to handle long range electrostatic interactions in reciprocal space. In all the three directions, periodic boundary conditions were imposed. Leapfrog algorithm was used to integrate the Newtonian equation of motion. The MD simulation was processed in an NVT ensemble and the simulation time is 500 ps. In NVT simulations, the temperature was maintained by the V-rescale thermostat at 298.15 K.

Computational Evaluation of Pore Size Distributions (PSD)

The theoretical pore size distributions (PSDs) of the extended multilayer framework models were computationally evaluated utilizing the Materials Studio software package. To rigorously quantify the sub-nanometer permeation bottlenecks, the spatial topology of the interconnected pore networks was mapped utilizing a grid-based Connolly surface algorithm. The accessible free volume was determined by evaluating the three-dimensional geometric void space defined by the hard-sphere van der Waals (vdW) boundaries of the framework atoms. Specifically, the scalar distance $d(\boldsymbol{r})$ from any spatial grid point $\boldsymbol{r}$ to the nearest framework atom is governed by this equation:

$$d(\boldsymbol{r}) = \min_i(|\boldsymbol{r} - \boldsymbol{R_i}| - r_{vdW,i})$$

In the equation, $\boldsymbol{R_i}$ is the spatial coordinate vector of atom $\boldsymbol{i}$, and $r_{vdW,i}$ represents its characteristic van der Waals radius assigned from the force field.

To extract the PSD, a test particle insertion (TPI) method integrated with a maximum inscribed sphere algorithm was employed across a finely discretized spatial grid (with a strict grid resolution of 0.15 Å). A spatial point $\boldsymbol{r}$ is geometrically deemed accessible to a specific kinetic probe of radius $\boldsymbol{r_p}$ if $d(\boldsymbol{r}) \geq \boldsymbol{r_p}$. The probability density function $f(D)$ of the local pore diameter D is subsequently derived and normalized across the

entire structural space according to this equation:

$$\int_0^\infty f(D)dD = 1$$

Prior to the volumetric integration, the structurally extended multilayer models underwent full geometric optimization to eliminate local high-energy structural overlaps. The resultant sub-nanometer PSD profiles mathematically represent the narrowest continuous kinetic bottlenecks formed within the complex 3D topological constrictions of the assembled macroscopic architecture, fundamentally defining the transmembrane size-sieving dimensions.

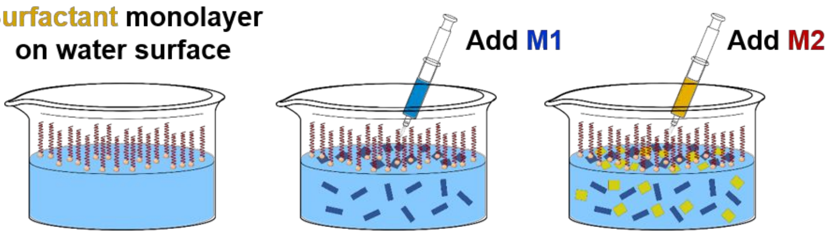

Figure S1. Schematic illustration of the synthesis of 2DPI using SMAIS method.

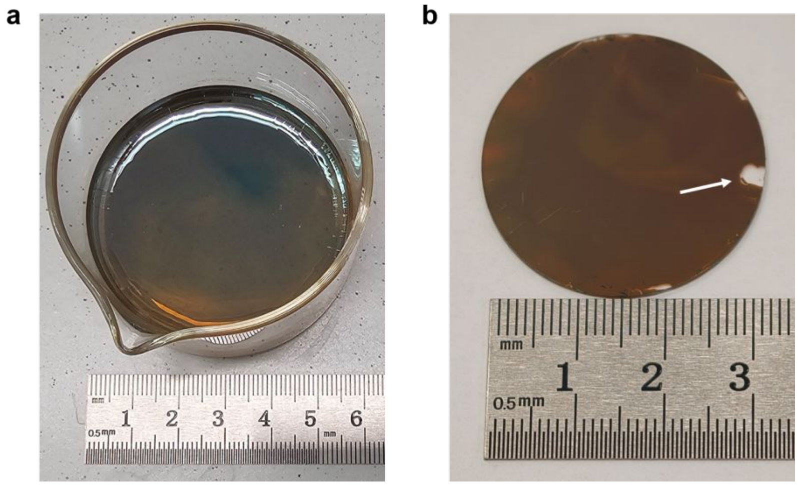

Figure S2. Macroscopic photographs demonstrating the scalability and processability of the Por-2DPI membranes. (a) A continuous, large-area ultrathin membrane synthesized via the SMAIS method in a crystallizing dish (diameter: 6 cm). (b) The membrane transferred onto a 3 cm diameter quartz substrate. The white arrow indicates a minor void at the edge caused by the tweezers during the transfer process. The slight wrinkles and micro-cracks at the edge normally occur due to capillary stress during solvent evaporation. Nevertheless, the large central region of the membrane maintains excellent uniformity, providing sufficient intact areas for the subsequent micro-area gas permeation measurements.

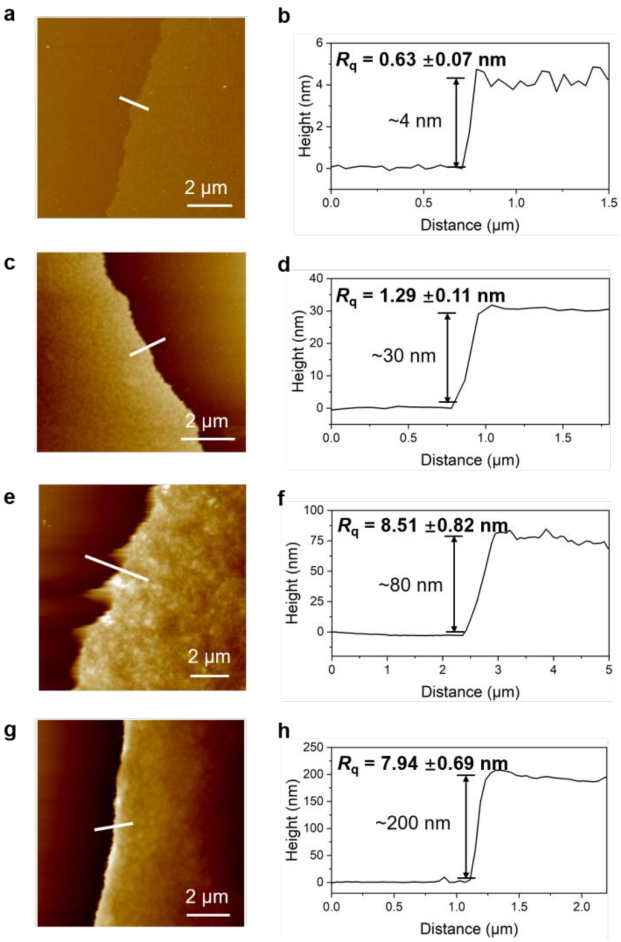

Figure S3. Quantitative surface roughness analysis of the Por-2DPI membranes at varying thicknesses. The 2D AFM topographic images in the left panels correspond to the exact same samples and regions shown in Figure 1d of the main text. The corresponding height profiles along the designated lines are presented in the right panels. The extracted root-mean-square roughness (R_q) values for the ~4, 30, 80, and 200 nm thick membranes are 0.63, 1.29, 8.51, and 7.94 nm, respectively. This non-linear evolution quantitatively confirms the transition from a highly confined smooth interface to an intermediate island-like aggregate morphology (~80 nm), followed by a macroscopic levelling effect via coalescence during prolonged growth (~200 nm).

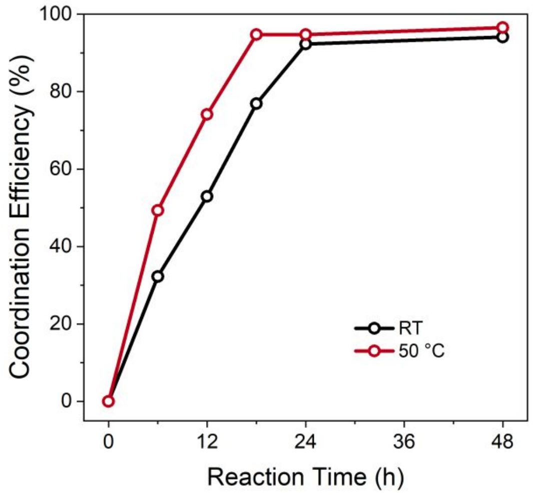

Figure S4. Optimization of the inner-pore coordination (IPC) conditions. Time-dependent coordination efficiency at room temperature (RT) and 50 °C, calculated quantitatively from the Cu/N atomic ratio using XPS analysis.

Optimization of IPC Reaction Time and Temperature. To evaluate the coordination kinetics, we quantitatively tracked the Cu/N atomic ratio using XPS across different reaction times (6 to 48 h) at both room temperature (RT) and 50 °C. At RT, the coordination efficiency exhibits a standard kinetic saturation profile. It steadily increases from 32.3% at 6 h to 76.9% at 18 h, reaching a plateau of 92.3% at 24 h. Extending the reaction to 48 h yields only a negligible increase (94.1%), indicating that the accessible coordination sites within the nanochannels are thermodynamically saturated within 24 h.

As expected, elevating the temperature to 50 °C accelerates the initial coordination rate (e.g., reaching 74.1% at 12 h compared to 52.9% at RT). However, the ultimate saturation limits at both temperatures are nearly identical (>92% after 24 h). Given that RT for 24 h is sufficient to achieve high coordination efficiency, it was adopted as the standard protocol. Maintaining RT simplifies the membrane fabrication process by avoiding additional thermal cycles and transfer steps, thereby minimizing the risk of mechanical perturbation or structural damage of the delicate ultra-thin COF films.

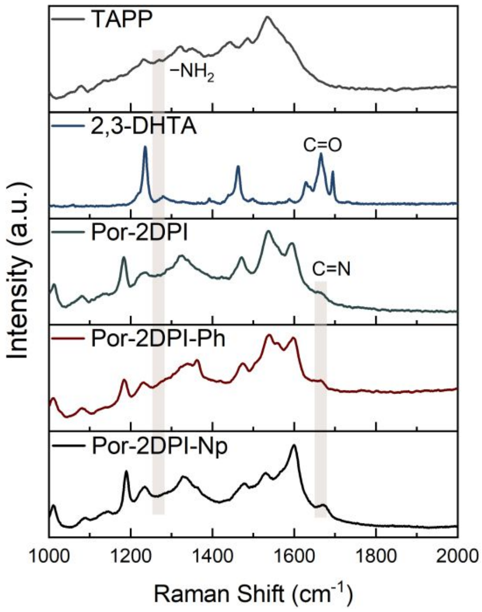

Figure S5. Raman spectra of precursor monomers and Por-2DPIs membranes.

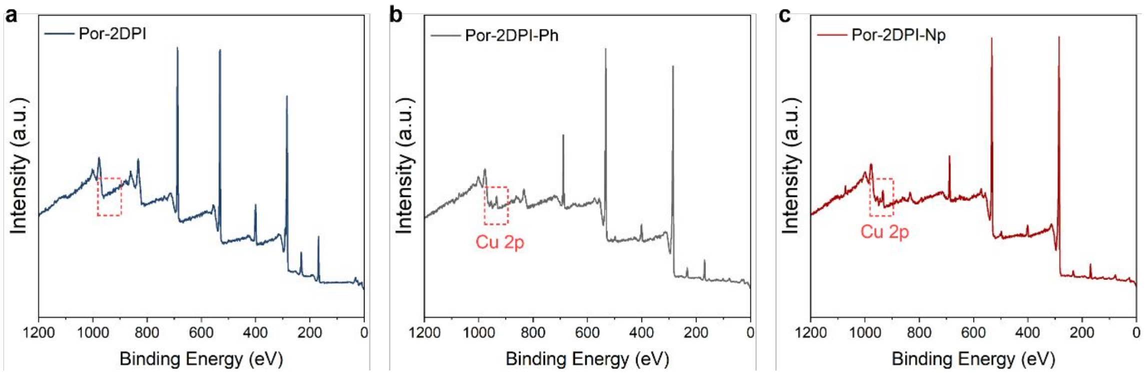

Figure S6. XPS survey spectra of Por-2DPI, Por-2DPI-Ph, and Por-2DPI-Np membranes.

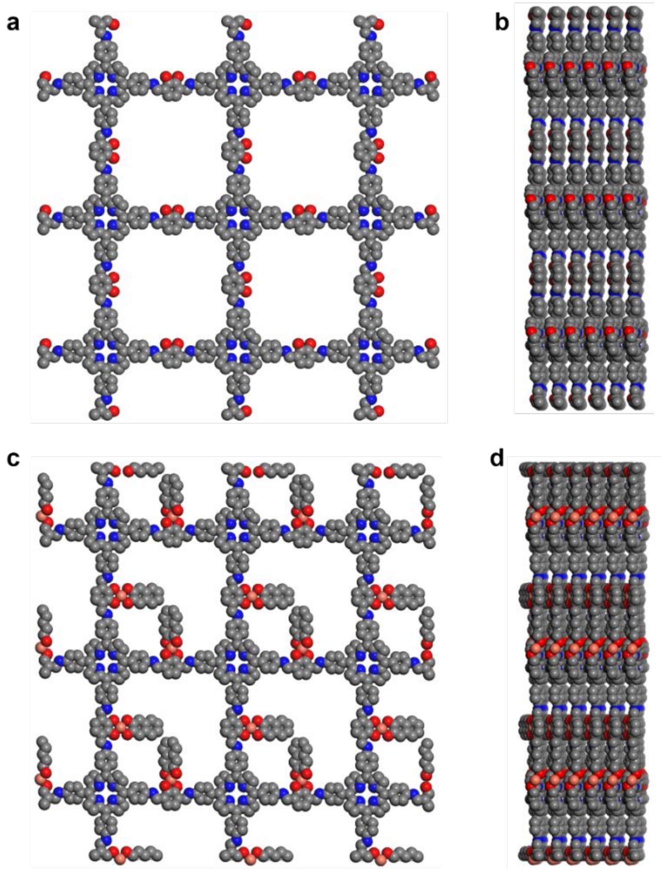

Figure S7. Calculated DFT structures of Por-2DPI (a,b) and Por- 2DPI-Np (c,d), respectively. Grey: carbon; blue: nitrogen; red: oxygen; white: hydrogen.

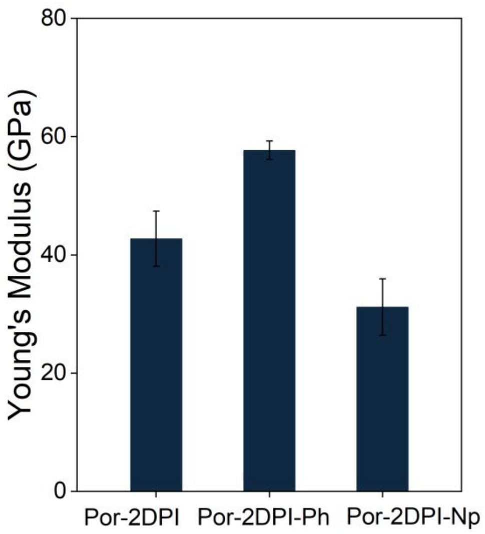

Figure S8. Nanoindentation measurements showing the Young's modulus of the freestanding Por-2DPI, Por-2DPI-Ph, and Por-2DPI-Np membranes (prepared via 6 transfer cycles, ~32 nm in thickness) suspended on bare copper grids.

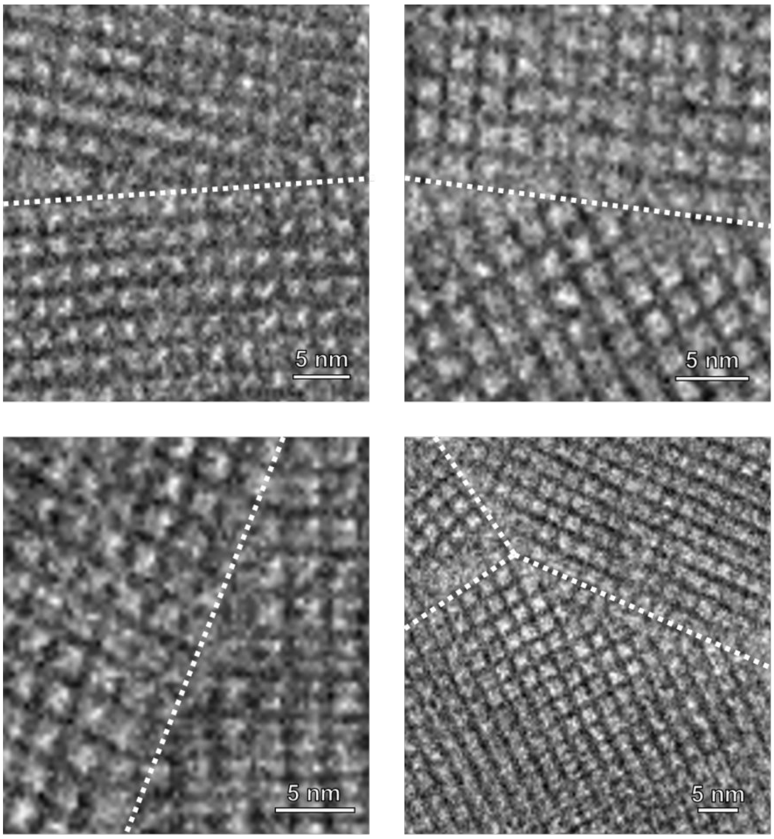

Figure S9. Grain boundaries of Por-2DPI observed under HRTEM (white dashed line).

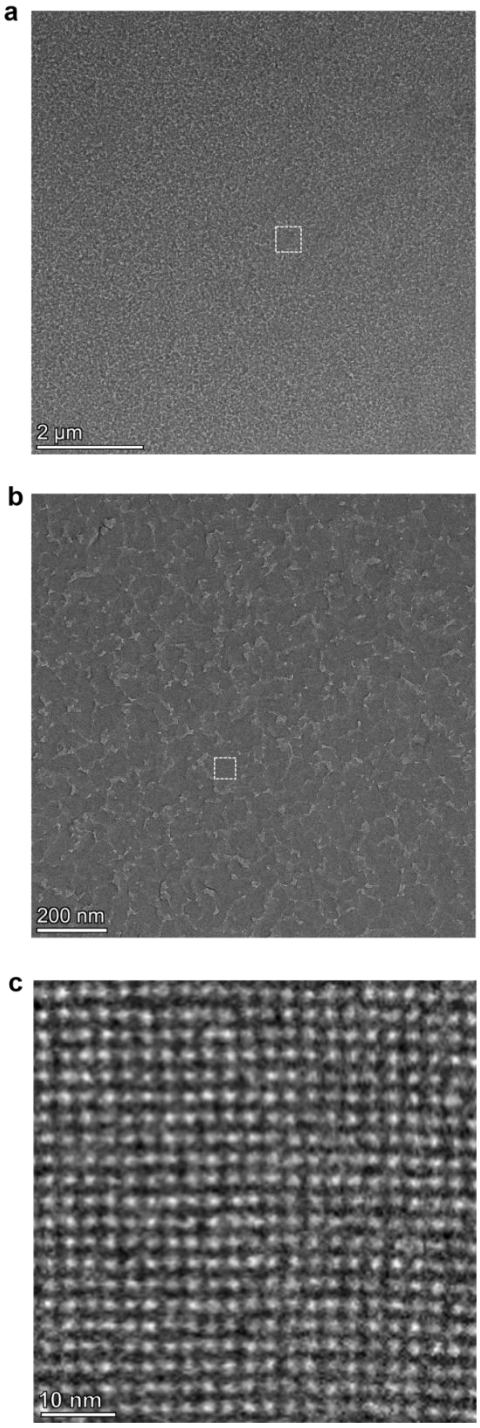

Figure S10. TEM images of Por-2DPI at the micrometer scale and higher magnification.

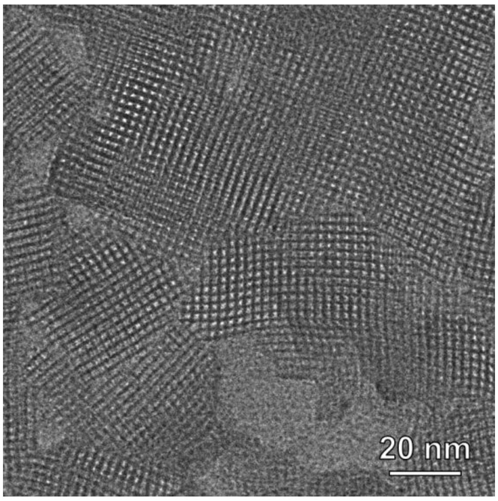

Figure S11. TEM image of Por-2DPI-Ph membrane.

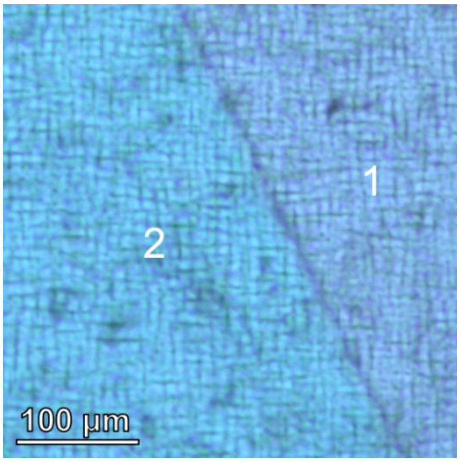

Figure S12. Optical microscopy image of Por-2DPI on PAN substrate (numbers 1 and 2 represent Por-2DPI of different layers)

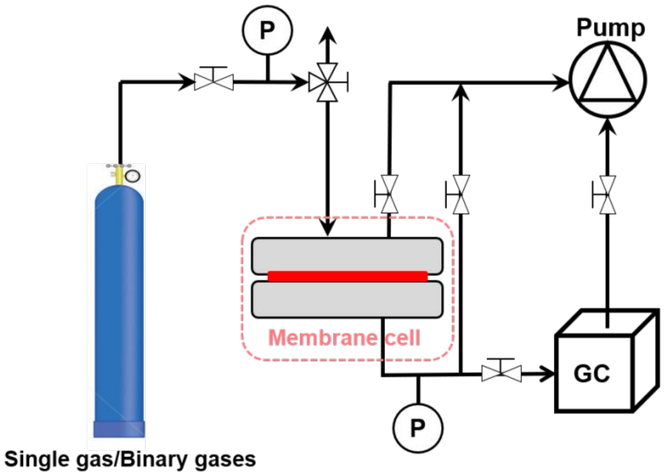

Figure S13. Schematic illustration of the home-made device for the permeation test of the membranes for pure gas and mixed gases.

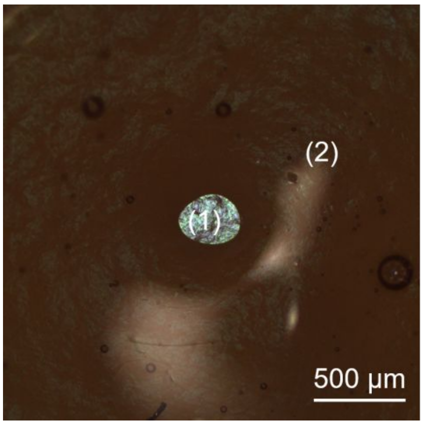

Figure S14. Representative optical microscopic image of the predefined aperture used for the micro-area gas permeation measurements. The central region **(1)** constitutes the effective testing area of the exposed Por-2DPI membrane, while the opaque peripheral region **(2)** represents the impermeable mask (aluminum foil tape and epoxy resin sealing). Based on strict calibration via image analysis, the precise effective membrane area for this specific sample is determined to be 0.063 mm^2.

For the ultra-thin COF membranes in this study with exceptionally high He permeance, the effective testing area was restricted using impermeable aluminum foil tape. This area restriction is a necessary instrumental adaptation to prevent the rapid saturation of the downstream pressure transducer, thereby ensuring a highly linear pressure accumulation (dp/dt) for accurate steady-state permeance derivation.

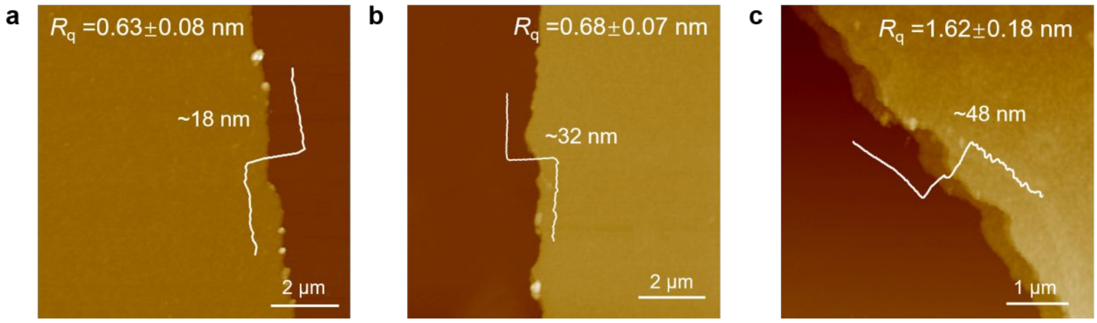

Figure S15. AFM images of the Por-2DPI membranes assembled via (a) 3, (b) 6, and (c) 9 transfer cycles. The white traces represent the AFM height profiles at selected positions.

The consistently low surface roughness maintained across multiple transfer cycles clearly indicates that the adjacent transferred layers are conformally and tightly adhered via van der Waals interactions, effectively minimizing severe macroscopic wrinkling or large interfacial voids.

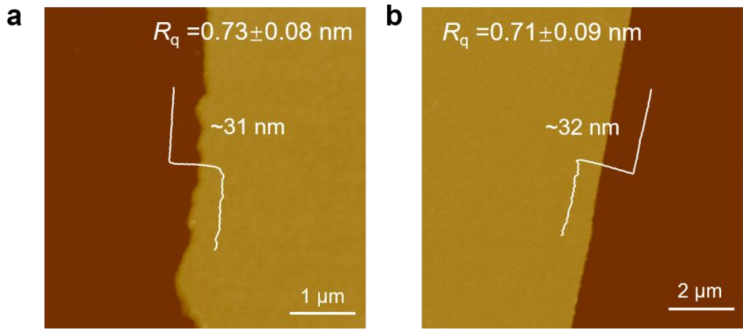

Figure S16. AFM images of the 6-layer (a) Por-2DPI-Ph and (b) Por-2DPI-Np membranes. The white traces represent the AFM height profiles at selected positions.

The ultra-smooth surfaces (e.g., $R_q \approx 0.70$ nm) confirm that the highly integrated, continuous macroscopic structure is perfectly preserved even after the inner-pore coordination modification.

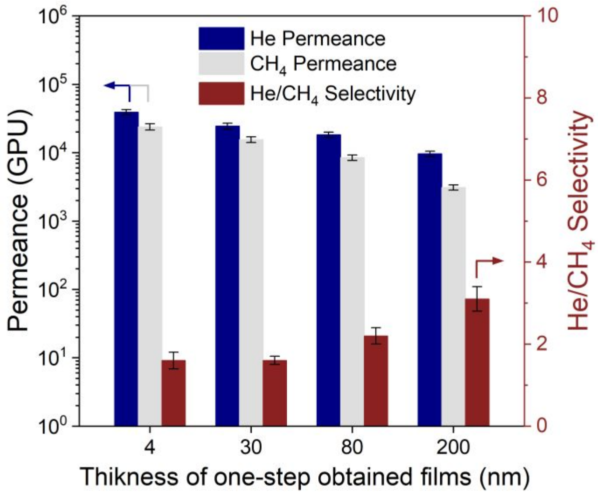

Figure S17. Thickness-dependent He/CH_4 separation performance of Por-2DPI membrane fabricated via *in situ* synthesis.

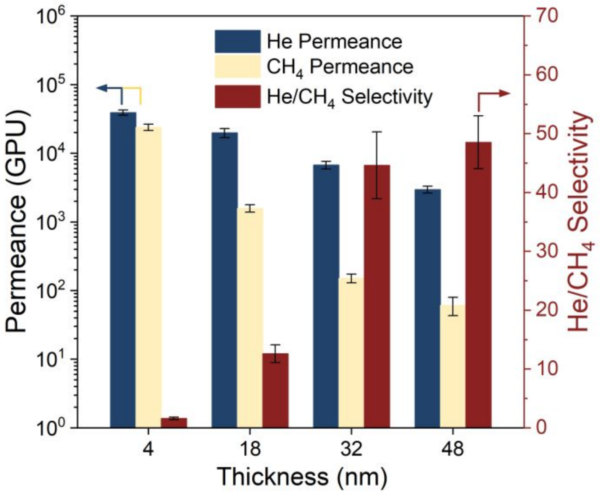

Figure S18. He/CH_4 separation performance of Por-2DPI membrane with tunable thickness achieved by layer-by-layer assembly.

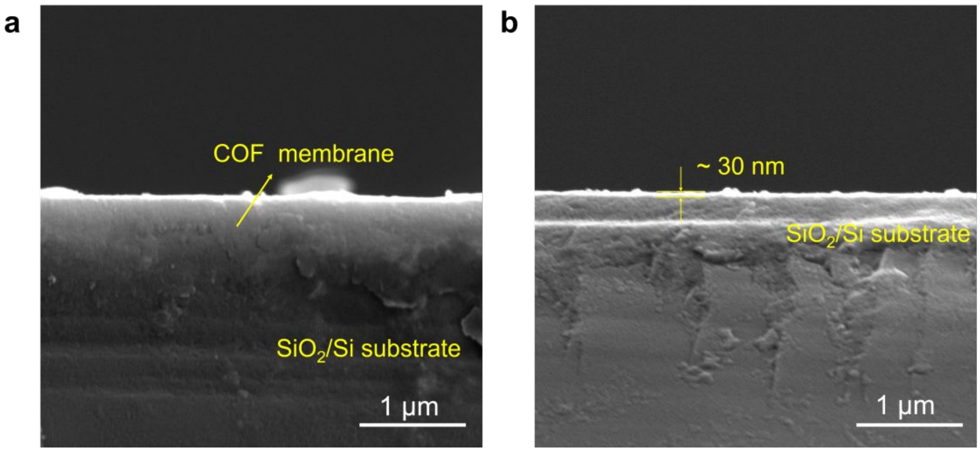

Figure S19. Cross-sectional microstructural characterizations of the 6-cycle Por-2DPI-Np membrane. (a) Cross-sectional SEM image captured at a mechanically disrupted edge, visualizing the continuous and robust nature of the ultrathin COF film (which partially overhangs due to the violent freeze-fracture process). (b) High-magnification cross-sectional SEM image in a flat fracture region, demonstrating the conformal adhesion of the ~30 nm COF layer onto the SiO_2/Si substrate without macroscopic voids. (Note: The bright contrast of the COF layer in (b) is attributed to the inherent electron-beam charging effect of the non-conducting ultrathin organic framework).

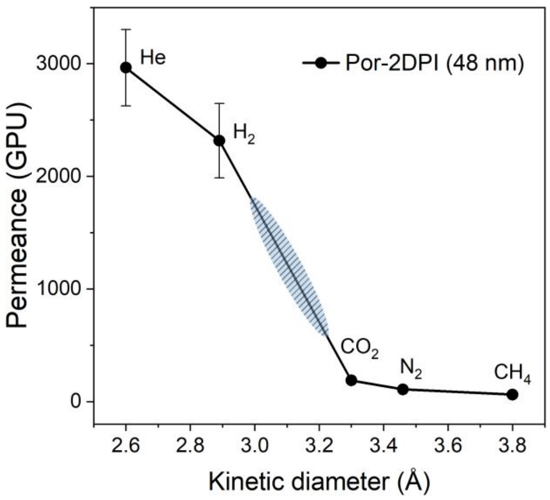

Figure S20. Single-gas permeances of the 48-nm-thick Por-2DPI membrane (assembled via 9 transfer cycles) as a function of the kinetic diameter of gas molecules. Compared to the 32-nm membrane (6 transfer cycles), increasing the thickness to 48 nm yields only a marginal enhancement in ideal He/CH_4 selectivity (from 44.6 to 48.5), but leads to a severe decline in absolute permeance (retaining only 44% of the He flux). This indicates that the pore-narrowing effect via physical staggered stacking experiences diminishing returns beyond 6 layers, making the 32-nm thickness an optimal balance between permeability and selectivity.

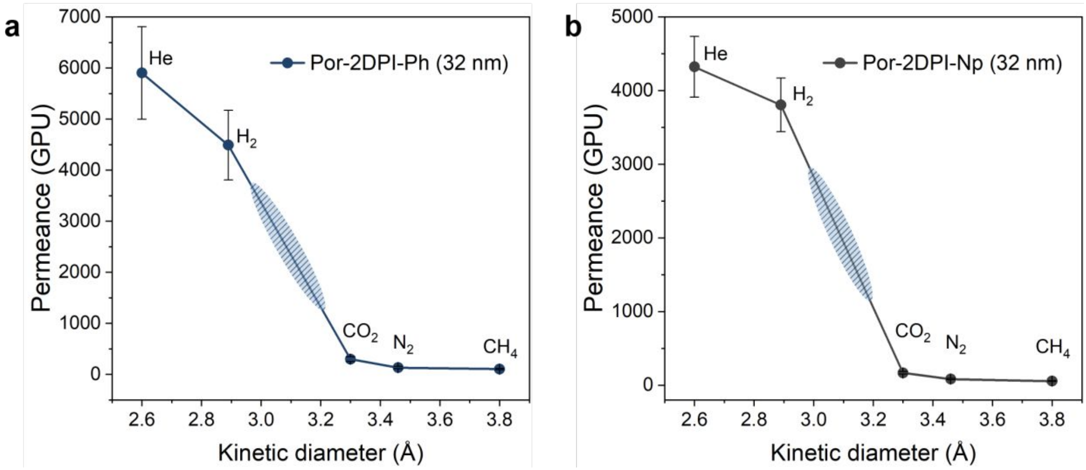

Figure S21. Single-gas permeances of the 32-nm-thick IPC-modified (a) Por-2DPI-Ph and (b) Por-2DPI-Np membranes as a function of the kinetic diameter of gas molecules. Following the IPC modification, the sub-nanometer bottlenecks within the channels are further refined. This specific chemical tailoring introduces additional steric hindrance predominantly for larger molecules (N_2 and CH_4), effectively elevating the He/ CH_4 and He/ N_2 selectivities while maintaining highly competitive He permeances.

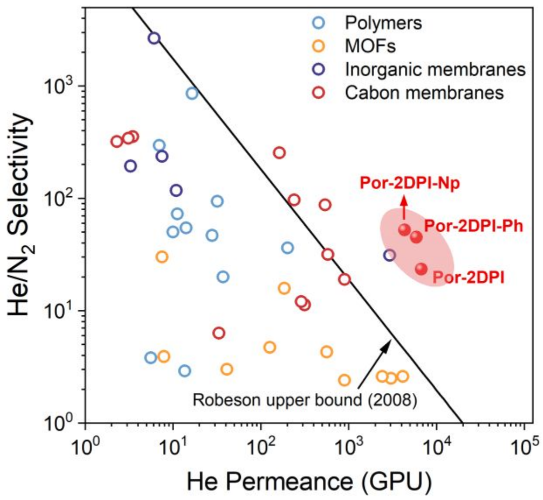

Figure S22. The performance comparison of He/N_2 separation between the prepared membranes in this work with other reported membranes in the literature.

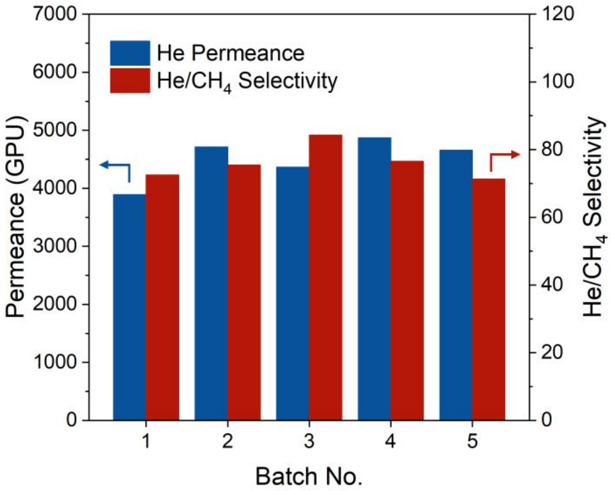

Figure S23. He permeance and He/CH_4 selectivity across 5 independent batches of Por-2DPI-Np membranes (from Table S2).

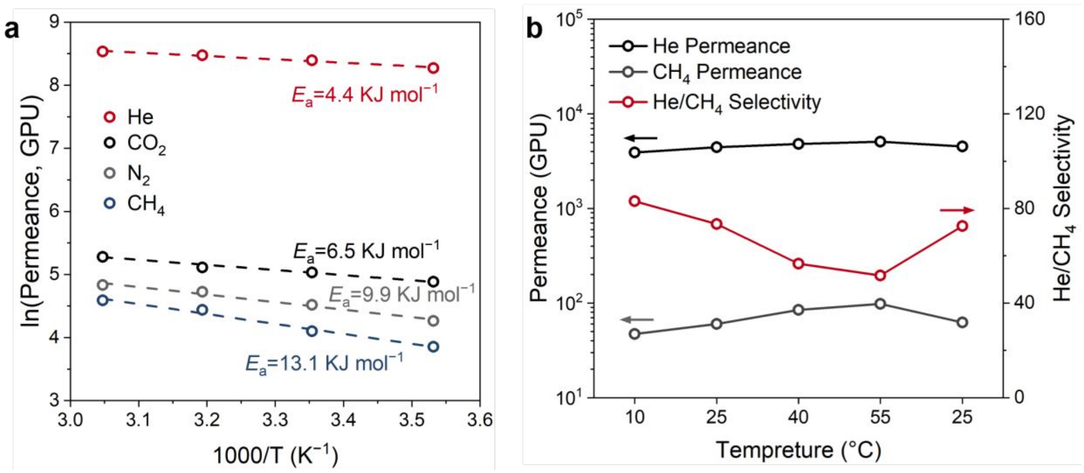

Figure S24. Temperature-dependent single-gas permeation performance of the Por-2DPI-Np membrane. (a) Arrhenius plots (lnP vs. 1000/T) for pure He, CO_2, N_2, and CH_4 measured from 10 to 55 °C. The extracted activation energies (E_a) strictly follow the sequence of the gas kinetic diameters. (b) Thermal cycling test evaluating the He and CH_4 permeance and the corresponding ideal selectivity. The testing temperature was incrementally elevated from 10 to 55 °C and subsequently cooled back to 25 °C. The fully reversible permeation behavior demonstrates the absence of structural degradation, confirming the excellent thermomechanical stability of the ultrathin membrane.

Calculation of Permeation Activation Energy

To fundamentally elucidate the transport kinetics, temperature-dependent gas permeation tests were conducted ranging from 10 to 55 °C. The relationship between gas permeance and temperature was analyzed using the classical Arrhenius equation:

$$P = P_0 \exp\left(\frac{-E_a}{RT}\right)$$

where P is the gas permeance, P_0 is the pre-exponential factor, E_a represents the apparent permeation activation energy, R is the ideal gas constant, and T is the absolute testing temperature. By plotting the natural logarithm of permeance ($ln\,P$) against the reciprocal of temperature (1000/T), the E_a values for each gas species were directly extracted from the slopes of the linear fits.

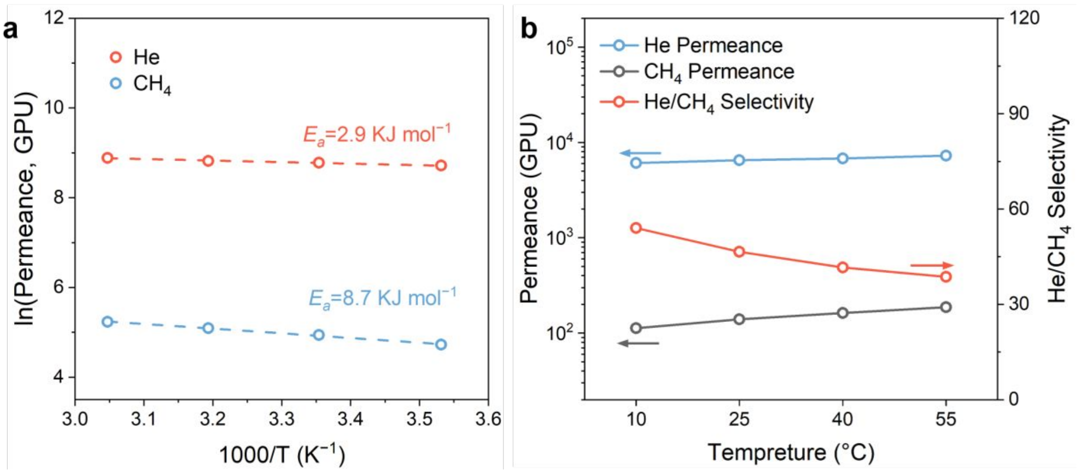

Figure S25. Temperature-dependent permeation performance of the pristine Por-2DPI membrane (baseline). (a) Arrhenius plots (ln P vs. 1000/T) for pure He and CH_4 measured from 10 to 55 °C. (b) Temperature-dependent He and CH_4 permeances and the corresponding ideal selectivity. Compared to the IPC-modified Por-2DPI-Np membrane (Figure S24), the pristine membrane exhibits a significantly lower transport barrier for CH_4 (8.7 vs. 13.1 kJ mol^{-1}). This baseline data explicitly confirms that while the intrinsic staggered stacking provides initial sieving, the IPC modification dramatically amplifies the steric hindrance to dominate the separation.

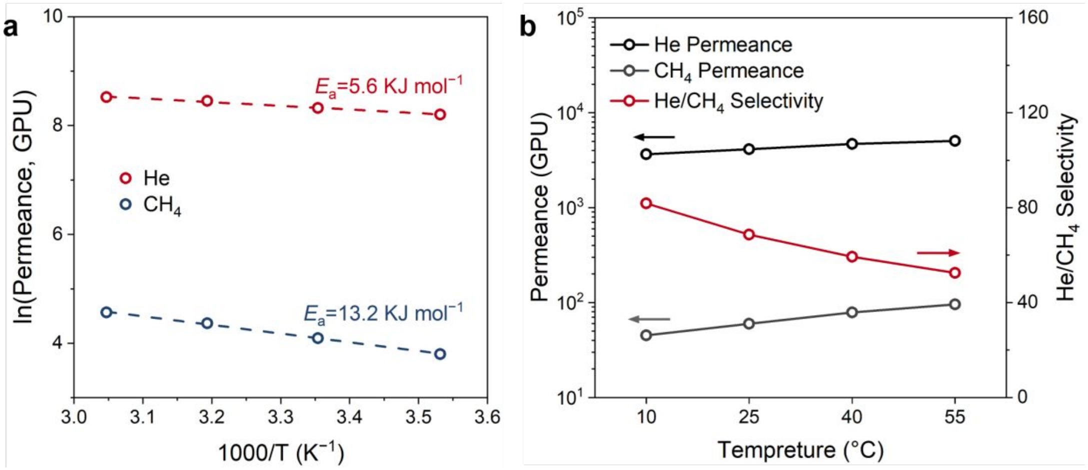

Figure S26. Temperature-dependent mixed-gas permeation performance of the Por-2DPI-Np membrane. (a) Arrhenius plots for He and CH_4 derived from an equimolar mixed-gas feed (50:50) from 10 to 55 °C. (b) Mixed-gas permeances and separation selectivity as a function of operating temperature. Compared to the single-gas kinetics, the E_a of He slightly increases due to the competitive hindrance from the slow-moving CH_4 within the confined channels. Nevertheless, a substantial kinetic gap (E_a: 5.6 vs. 13.2 kJ mol^{-1}) is robustly preserved, verifying the stability of the rigid size-sieving mechanism under competitive separation conditions.

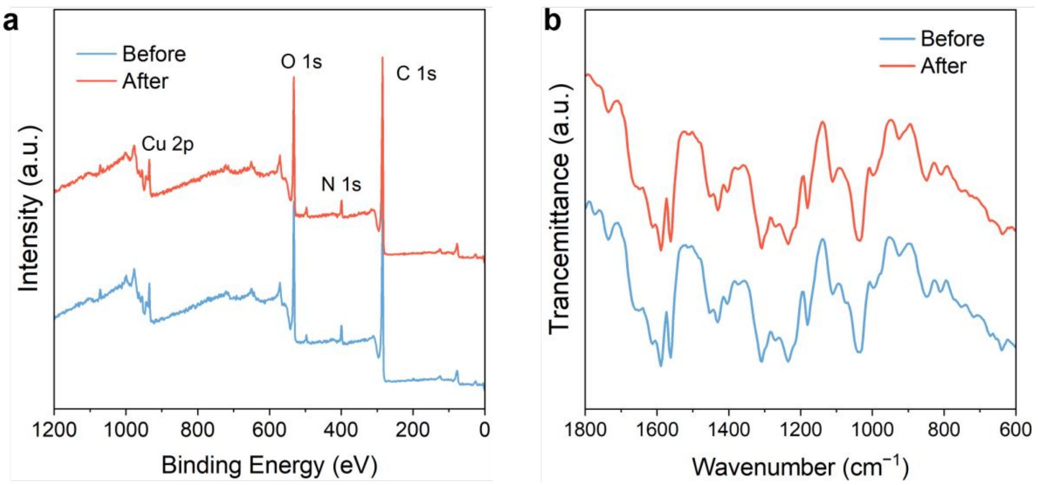

Figure S27. Post-test ex-situ microstructural characterizations of the Por-2DPI-Np (PAN) membrane. (a) XPS survey and (b) ATR-FTIR spectra of the membrane before and after the long-term mixed-gas permeation test. The nearly identical spectral profiles explicitly confirm that the intrinsic chemical states, coordination sites, and covalent framework integrity are robustly preserved without discernible degradation under prolonged transmembrane pressure.

This operational stability arises from three structural features: (1) the rigid polyimine network and interlayer π-π interactions resist chemical degradation from continuous gas flow; (2) the staggered staggered overlapping architecture dissipates localized mechanical stress, preventing microcrack propagation; and (3) mild thermal annealing promotes van der Waals adhesion between the COF film and the PAN support, securing macroscopic integrity.

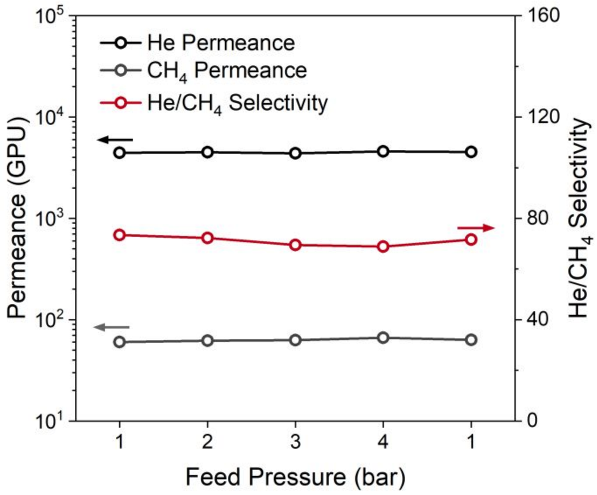

Figure S28. Pressure-dependent single-gas permeation and stability of the Por-2DPI-Np membrane. Permeances of pure He and CH_4, and the corresponding ideal He/CH_4 selectivity measured at 25 °C as a function of the feed pressure. The membrane was subjected to an escalating pressure ramp from 1 to 4 bar, followed by a direct depressurization back to 1 bar. The stable permeances across this pressure range exclude viscous flow through macroscopic defects and adsorption-enhanced transport. The reversible separation performance after the pressure cycle rules out pressure-induced structural yielding confirming the structural resilience of the size-sieving channels.

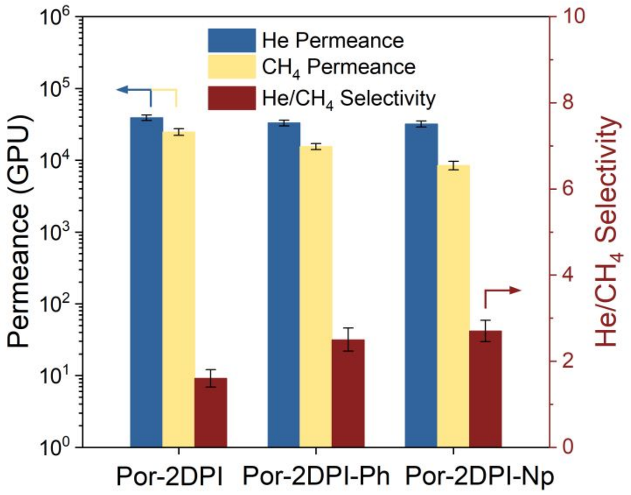

Figure S29. Single-gas He/CH_4 separation performance of the single-layer (~4 nm) COF membranes. Single-gas permeances through the 1-transfer-cycle Por-2DPI, Por-2DPI-Ph, and Por-2DPI-Np membranes as a function of the kinetic diameter at 1 bar and 25 °C. While all three ultra-thin membranes exhibit massive absolute permeances, their ideal selectivities remain broadly unselective (approaching the theoretical Knudsen diffusion limits), irrespective of the IPC modification. This control experiment explicitly demonstrates that chemical pore-narrowing within a discrete ultrathin 1-cycle film is insufficient for precise gas sieving. It strictly corroborates that the high selectivity observed in the optimized 6-cycle membranes relies on a structural-chemical synergy: the coupling of IPC-tailored kinetic bottlenecks with the highly tortuous transmembrane pathways generated by vertical staggered stacking.

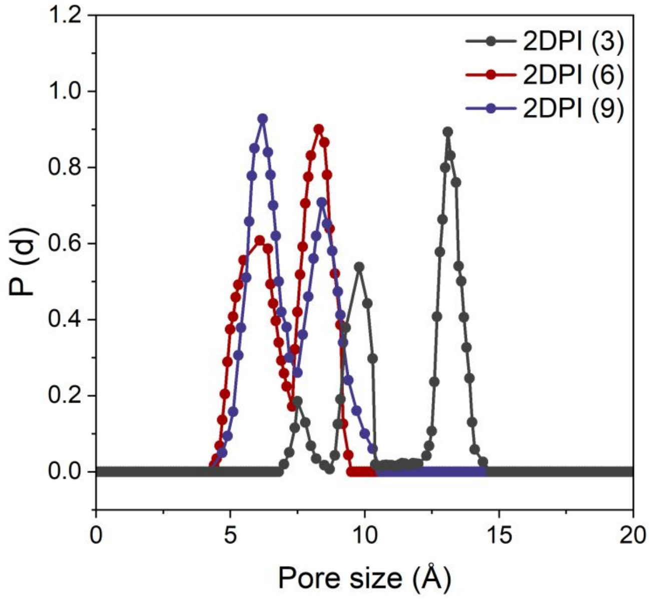

Figure S30. Pore size distribution of Por-2DPI stacked 3, 6 and 9 layers.

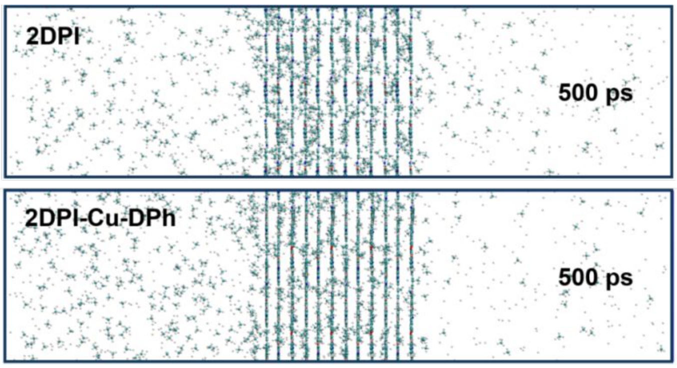

Figure S31. Simulation system with snapshot at 500 ps for the permeation of equimolar helium and methane through 2DPI and 2DPI-Cu-DPh.

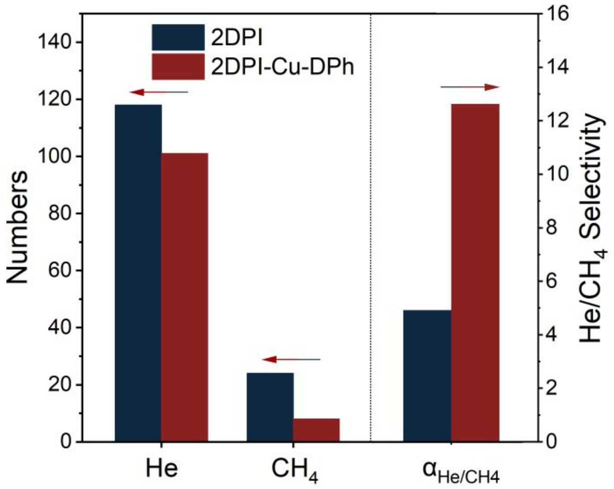

Figure S32. The numbers and selectivity of gas molecules passing through 2DPI and 2DPI-Cu-DPh films in 200 ps through molecules dynamic (MD) simulation.

Table S1. A summary of the He/CH_4 and He/N_2 separation performance of membranes in reported literature.

	Membranes	T (°C)	P (bar)	Gas Type	P_{He} (GPU)	He/CH_4	He/N_2	Ref.
	Por-2DPI	25	1	single	6727	44.6	23.4	This work
	Por-2DPI-Ph	25	1	single	5902	56.8	44.7	
	Por-2DPI-Np	25	1	single	4323	77.4	52.3	
MOFs	Cu-BTC	25	1	single	4140.0	2.1	2.6	5
	IRMOF-3	25	1	single	3046.0	1.6	2.5	6
	IRMOF-3-AM6	25	1	single	2416.0	1.3	2.6	
	MMOF	25	1	single	41.0	–	3.0	7
	MMOF	190	1	single	7.5	–	30.0	
	$[Cu_2(bza)_4(pyz)_n]$	20	–	single	7.9	7.3	3.9	8
	MOF-5	25	1.7	single	896.0	–	2.4	9
	ZIF-8	25	1	single	565.0	4.6	4.3	10
	ZIF-8	35	1	single	126.6	4.0	4.7	11
	3.50%C_{70}@ZIF-8	35	1	single	185.4	9.2	15.8	12
Polymers	Nafion-117	35	0.4	single	32.0	56.3	94.0	13
	Poly(PFMD)	35	3.4	single	7.0	1650.0	295.8	14
	Poly(PFMMD)	35	3.4	single	11.2	280.0	72.7	14
	Cellulose acetate	22	4.5	single	28.0	40.0	46.7	15
	Polysulphone	35	2	single	0.5	49.0	52.0	16
	STT	25	1	single	37.1	87.0	20.0	17
	SAPO-34	25	1.4	mixed	554.1	13.8	–	18
	DD3R	25	1	single	13.6	79.0	2.9	19
	TR-6FDA-APAF-Cardo	25	1	mixed	14.1	64.0	54.4	20

	TR-6FDA-APAF$_{0.5}$-Cardo$_{0.5}$/Al_2O_3	25	1	mixed	10.0	71.2	50.0	20, 21
	TR-6FDA-APAF	35	1	single	2.4	37.2	–	
	PIM-EA-TB	25	1	single	14. 2	3.7	4.9	22
	PIM-SBI-TB	25	1	single	5.6	2.0	3.8	22
	Fluorinated PIM	35	1	single	202.0	61.2	36.1	23
	FPIM-5	35	-	single	16.6	3770.0	857	24
Carbon membranes	polyimide/silica	25	1	single	163.4	837.1	253.8	25
		35	1	single	239.2	187.1	96.7	
	sulfonated phenolic resin	35	1	single	539.0	201.9	87.3	26
		35	1	single	581.7	41.8	31.6	
		35	1	single	895.8	30.1	19.0	
	metal oxide/PIM-PI	35	2	single	316.2	11.3	11.3	27
		35	2	single	288.8	12.7	12.0	
		60	1	single	33.4	5.0	6.3	
	P84	60	1	single	3.5	415.3	353.0	28
		60	1	single	3.1	603.9	342.2	
		60	1	single	2.3	2925.0	320.5	
Inorganic membranes	Microporous Silica	35	0.5	single	2933.0	147.0	31.0	29
	TESO-derived Silica	200	2	single	3.3	1095.7	193.4	30
	F−SiO_2 with F/Si ratio: 2/8	35	–	single	10.9	272.8	117.3	31
	Silica derived from DMDMS	500	1	single	6.1	2772.7	2652.2	32
	VTES-derived silica	300	2.5	single	7.5	668.4	236.7	33

Table S2. Structural parameters and gas separation performance of Por-2DPI-Np membranes across five independent batches.

Batch No.	Transfer Cycles	Thickness[a] (nm)	Effective Area[b] (mm^2)	He Permeance (GPU)	Ideal Selectivity (He/CH_4)
1	6	32	0.038	3893	72.5
2	6	32	0.033	4714	75.5
3	6	31	0.019	4365	84.3
4	6	31	1.28	4869	76.6
5	6	32	5.76	4658	71.3

[a] Determined by AFM measurements on parallelly co-transferred Si/SiO_2 substrates from the same synthesis batch.

[b] The actual exposed membrane area on the porous PAN substrate used for gas permeation testing.

To evaluate the structural and separation consistency, we systematically investigated five independent batches of the Por-2DPI-Np membranes. The membrane thickness remained highly uniform at approximately 32 nm, confirming the precision of the layer-by-layer SMAIS assembly. Gas permeation tests further demonstrated excellent performance reproducibility; the He permeance averaged ~4300 GPU and the He/CH_4 selectivity was robustly maintained at ~76.0 with a relative standard deviation of less than 10%. Notably, this separation integrity was preserved even when the effective testing area was scaled up nearly 300-fold (from 0.019 to 5.76 mm^2), confirming that the IPC strategy effectively suppresses macroscopic defect formation across different batches and dimensions.

Table S3. Separation performance of Por-2DPIs membranes at room temperature and 1 bar for single gas and equimolar binary gases.

Polymer	Single gas		Mixed gas	
	P_{He}	$\alpha_{He/CH4}$	P_{He}	$\alpha_{He/CH4}$
Por-2DPI (32 nm)	6727	44.6	6408	40.9
Por-2DPI-Ph (31 nm)	5902	56.8	5351	51.3
Por-2DPI-Np (32 nm)	4323	77.4	4171	74.3

References

(1) Cheng, B.; Zhong, Y.; Qiu, Y.; Vaikuntanathan, S.; Park, J., Giant Gateable Osmotic Power Generation from a Goldilocks Two-Dimensional Polymer. *J. Am. Chem. Soc.* **2023,** *145* (9), 5261-5269.

(2) Meng, Q. W.; Zhu, X. C.; Xian, W. P.; Wang, S.; Zhang, Z. Q.; Zheng, L. P.; Dai, Z. F.; Yin, H.; Ma, S. Q.; Sun, Q., Enhancing Ion Selectivity by Tuning Solvation Abilities of Covalent-Organic-Framework Membranes. *Proc. Natl. Acad. Sci. U.S.A.* **2024,** *121* (8), e2316716121.

(3) Jorgensen, W. L.; Maxwell, D. S.; TiradoRives, J., Development and testing of the OPLS all-atom force field on conformational energetics and properties of organic liquids. *J. Am. Chem. Soc.* **1996,** *118* (45), 11225-11236.

(4) Darden, T. A.; York, D. M.; Pedersen, L. G., Particle Mesh Ewald: An N·log(N) Method for Ewald Sums in Large Systems. *J. Chem. Phys.* **1993,** *98*, 10089-10092.

(5) Cao, F.; Zhang, C. J.; Xiao, Y. L.; Huang, H. L.; Zhang, W. J.; Liu, D. H.; Zhong, C. L.; Yang, Q. Y.; Yang, Z. H.; Lu, X. H., Helium Recovery by a Cu-BTC Metal-Organic-Framework Membrane. *Ind. Eng. Chem. Res.* **2012,** *51* (34), 11274-11278.

(6) Yoo, Y.; Varela-Guerrero, V.; Jeong, H. K., Isoreticular Metal-Organic Frameworks and Their Membranes with Enhanced Crack Resistance and Moisture Stability by Surfactant-Assisted Drying. *Langmuir* **2011,** *27* (6), 2652-2657.

(7) Ranjan, R.; Tsapatsis, M., Microporous Metal Organic Framework Membrane on Porous Support Using the Seeded Growth Method. *Chem. Mater.* **2009,** *21* (20), 4920-4924.

(8) Takamizawa, S.; Takasaki, Y.; Miyake, R., Single-Crystal Membrane for Anisotropic and Efficient Gas Permeation. *J. Am. Chem. Soc.* **2010,** *132* (9), 2862-+.

(9) Zhao, Z. X.; Ma, X. L.; Li, Z.; Lin, Y. S., Synthesis, Characterization and Gas Transport Properties of MOF-5 Membranes. *J. Membr. Sci.* **2011,** *382* (1-2), 82-90.

(10) Liu, D.; Ma, X.; Xi, H.; Lin, Y., Gas Transport Properties and Propylene/Propane Separation Characteristics of ZIF-8 Membranes. *J. Membr. Sci.* **2014,** *451*, 85-93.

(11) Hara, N.; Yoshimune, M.; Negishi, H.; Haraya, K.; Hara, S.; Yamaguchi, T., Diffusive Separation of Propylene/Propane with ZIF-8 Membranes. *J. Membr. Sci.*

2014, *450*, 215-223.

(12) Han, J. L.; Wu, H. Y.; Fan, H. W.; Ding, L.; Hai, G. T.; Caro, J.; Wang, H. H., Tuning the Phase Composition of Metal-Organic Framework Membranes for Helium Separation through Incorporation of Fullerenes. *J. Am. Chem. Soc.* **2023,** *145* (27), 14793-14801.

(13) Choi, S. H.; Qahtani, M. S.; Qasem, E. A., Multilayer Thin-Film Composite Membranes for Helium Enrichment. *J. Membr. Sci.* **2018,** *553*, 180-188.

(14) Yavari, M.; Fang, M. F.; Nguyen, H.; Merkel, T. C.; Lin, H. Q.; Okamoto, Y., Dioxolane-Based Perfluoropolymers with Superior Membrane Gas Separation Properties. *Macromolecules* **2018,** *51* (7), 2489-2497.

(15) Gantzel, P. K.; Merten, U., Gas Separations with High-Flux Cellulose Acetate Membranes. *Ind. Eng. Chem. Process Des. Dev.* **1970,** *9* (2), 331-&.

(16) McHattie, J. S.; Koros, W. J.; Paul, D. R., Gas-Transport Properties of Polysulfones. 2. Effect of Bisphenol Connector Groups. *Polymer* **1991,** *32* (14), 2618-2625.

(17) Gong, C.; Peng, X. Y.; Zhu, M. Y.; Zhou, T.; You, L.; Ren, S. Y.; Wang, X. R.; Gu, X. H., Synthesis and Performance of STT Zeolite Membranes for He/N_2 and He/CH_4 Separation. *Sep. Purif. Technol.* **2022,** *301*.

(18) Denning, S.; Lucero, J.; Koh, C. A.; Carreon, M. A., Chabazite Zeolite SAPO-34 Membranes for He/CH_4 Separation. *ACS Mater. Lett.* **2019,** *1* (6), 655-659.

(19) Zhang, P.; Gong, C.; Zhou, T.; Du, P.; Song, J. Y.; Shi, M. Y.; Wang, X. R.; Gu, X. H., Helium Extraction from Natural Gas Using DD3R Zeolite Membranes. *Chin. J. Chem. Eng.* **2022,** *49*, 122-129.

(20) Wang, L.; Li, Y.; Zhang, P.; Chen, X. F.; Nian, P.; Wei, Y. B.; Lu, H. S.; Gu, X. H.; Wang, X. R., Thermally Rearranged Poly(benzoxazole-co-imide) Composite Membranes on α-Al_2O_3 Support for Helium Extraction from Natural Gas. *J. Membr. Sci.* **2022,** *657*.

(21) Calle, M.; Lee, Y. M., Thermally Rearranged (TR) Poly(ether-benzoxazole) Membranes for Gas Separation. *Macromolecules* **2011,** *44* (5), 1156-1165.

(22) Carta, M.; Malpass-Evans, R.; Croad, M.; Rogan, Y.; Jansen, J. C.; Bernardo, P.;

Bazzarelli, F.; McKeown, N. B., An Efficient Polymer Molecular Sieve for Membrane Gas Separations. *Science* **2013,** *339* (6117), 303-307.
(23) Seong, J. G.; Lee, W. H.; Lee, J.; Lee, S. Y.; Do, Y. S.; Bae, J. Y.; Moon, S. J.; Park, C. H.; Jo, H. J.; Kim, J. S.; Lee, K. R.; Hung, W. S.; Lai, J. Y.; Ren, Y.; Roos, C. J.; Lively, R. P.; Lee, Y. M., Microporous Polymers with Cascaded Cavities for Controlled Transport of Small Gas Molecules. *Sci. Adv.* **2021,** *7* (40).
(24) Ma, X. H.; Li, K. H.; Zhu, Z. Y.; Dong, H.; Lv, J.; Wang, Y. G.; Pinnau, I.; Li, J. X.; Chen, B. W.; Han, Y., High-Performance Polymer Molecular Sieve Membranes Prepared by Direct Fluorination for Efficient Helium Enrichment. *J. Mater. Chem. A* **2021,** *9* (34), 18313-18322.
(25) Park, H. B.; Lee, Y. M., Fabrication and Characterization of Nanoporous Carbon/Silica Membranes. *Adv. Mater.* **2005,** *17* (4), 477-+.
(26) Zhou, W. L.; Yoshino, M.; Kita, H.; Okamoto, K., Preparation and Gas Permeation Properties of Carbon Molecular Sieve Membranes Based on Sulfonated Phenolic Resin. *J. Membr. Sci.* **2003,** *217* (1-2), 55-67.
(27) Ogieglo, W.; Puspasari, T.; Hota, M. K.; Wehbe, N.; Alshareef, H. N.; Pinnau, I., Nanohybrid Thin-Film Composite Carbon Molecular Sieve Membranes. *Mater. Today Nano.* **2020,** *9*.
(28) Favvas, E. P.; Heliopoulos, N. S.; Papageorgiou, S. K.; Mitropoulos, A. C.; Kapantaidakis, G. C.; Kanellopoulos, N. K., Helium and Hydrogen Selective Carbon Hollow Fiber Membranes: The Effect of Pyrolysis Isothermal Time. *Sep. Purif. Technol.* **2015,** *142*, 176-181.
(29) Asaeda, M.; Yamasaki, S., Separation of Inorganic/Organic Gas Mixtures by Porous Silica Membranes. *Sep. Purif. Technol.* **2001,** *25* (1-3), 151-159.
(30) ten Hove, M.; Luiten-Olieman, M. W. J.; Huiskes, C.; Nijmeijer, A.; Winnubst, L., Hydrothermal Stability of Silica, Hybrid Silica and Zr-Doped Hybrid Silica Membranes. *Sep. Purif. Technol.* **2017,** *189*, 48-53.
(31) Kanezashi, M.; Matsutani, T.; Wakihara, T.; Tawarayama, H.; Nagasawa, H.; Yoshioka, T.; Okubo, T.; Tsuru, T., Tailoring the Subnano Silica Structure via Fluorine Doping for Development of Highly Permeable CO_2 Separation Membranes.

Chemnanomat **2016,** *2* (4), 264-267.

(32) Akamatsu, K.; Suzuki, M.; Nakao, A.; Nakao, S., Development of Hydrogen-Selective Dimethoxydimethylsilane-Derived Silica Membranes with Thin Active Separation Layer by Chemical Vapor Deposition. *J. Membr. Sci.* **2019,** *580*, 268-274.

(33) Ahn, S. J.; Yun, G. N.; Takagaki, A.; Kikuchi, R.; Oyama, S. T., Synthesis and Characterization of Hydrogen Selective Silica Membranes Prepared by Chemical Vapor Deposition of Vinyltriethoxysilane. *J. Membr. Sci.* **2018,** *550*, 1-8.
